# SPP1 as a Critical Regulator of Cardiac Cell Reprogramming Following Myocardial Infarction Through Single‐Cell Transcriptomic Analysis

**DOI:** 10.1155/humu/3656018

**Published:** 2026-04-17

**Authors:** Rui Wang, Man Zhang, Xiaojun Liu, Zi Li, Xueqing Chen

**Affiliations:** ^1^ Cardiovascular Department, Xiongan Xuanwu Hospital, Xiongan New District, China; ^2^ Cardiac Macrovascular Surgery Ward, Zibo Central Hospital, Zibo City, China, zbzxyy.com

**Keywords:** cardiac cell reprogramming, computational biology, myocardial infarction, regenerative medicine, single-cell transcriptomics, SPP1 biomarker

## Abstract

**Background:**

Cardiovascular mortality remains predominantly driven by acute myocardial infarction (AMI), necessitating comprehensive elucidation of mechanisms governing cardiomyocyte reprogramming for therapeutic advancement. Characterizing molecular dynamics throughout cardiac repair processes presents substantial methodological challenges.

**Methods:**

We employed a comprehensive analytical framework combining bulk and single‐cell RNA sequencing datasets from AMI patients, utilizing 26 distinct machine learning algorithms to delineate critical regulatory gene signatures associated with cardiac repair. Gene prioritization was achieved through differential expression profiling coupled with consensus clustering methodologies. Functional validation experiments in H9c2 cardiomyoblast cellular models demonstrated that enhanced SPP1 expression is associated with augmented cellular viability and stimulated cardioprotective factor release, though these findings require validation in more physiologically relevant systems.

**Results:**

Machine learning architectures successfully identified robust cardiomyocyte reprogramming signatures correlating with cardiac functional restoration. Consensus clustering analysis of 276 genes revealed two phenotypically distinct repair subtypes demonstrating divergent recovery trajectories (*p* < 0.001). Enhanced‐recovery clusters exhibited upregulated proliferation markers alongside intensified angiogenic signaling cascades. SPP1 demonstrated exceptional predictive capacity (AUC = 0.896), with experimental validation suggesting its potential functional role in promoting cellular survival, proliferation, and secretion of cardioprotective mediators (VEGF, IGF‐1; all *p* < 0.05).

**Conclusion:**

This machine learning–driven approach successfully identified novel candidate prognostic biomarkers and potential therapeutic targets for cardiac repair interventions, substantially advancing mechanistic understanding of postinfarction cardiac remodeling processes. These findings generate testable hypotheses requiring validation in independent clinical cohorts and more physiologically relevant experimental systems.

## 1. Introduction

Cardiovascular medicine confronts persistent challenges posed by acute myocardial infarction (AMI), where elevated mortality stems primarily from irreversible cardiomyocyte depletion combined with inherent heterogeneity characterizing cardiac repair mechanisms [1–3]. Recent therapeutic innovations, including regenerative interventions and cellular cardiac therapies, have fundamentally transformed approaches to managing postinfarction cardiac remodeling. However, heterogeneous patient responses to regenerative therapeutics, coupled with the multifaceted architecture of the cardiac repair microenvironment, substantially complicate optimal patient stratification and therapeutic planning [4–6].Contemporary investigations into the cardiac repair microenvironment have illuminated the pivotal contribution of cardiomyocyte reprogramming to both AMI prognosis and therapeutic efficacy. This microenvironment represents a heterogeneous, dynamically evolving ecosystem comprising cardiomyocytes, fibroblasts, endothelial populations, and inflammatory cells engaging in multifaceted intercellular communication networks that modulate cardiac remodeling, functional restoration, and therapeutic responsiveness. The compositional diversity and functional heterogeneity of cardiac cells populating the repair microenvironment have emerged as principal determinants of patient prognosis [7–9]. Despite mounting evidence substantiating the significance of cardiomyocyte reprogramming in AMI pathophysiology, precise quantification and interpretation of the cardiac repair landscape at single‐cell resolution remain constrained. Conventional methodologies prove inadequate for capturing the dynamic complexity of cell–cell interactions owing to pronounced heterogeneity among cardiac cell populations. Machine learning (ML) emerges as an invaluable analytical tool for deciphering intricate cardiomyocyte reprogramming signatures in AMI contexts. ML algorithms possess the capability to process extensive multisource datasets (encompassing genomics, transcriptomics, and proteomics) with the objective of identifying complex relational and predictive patterns that might elude conventional detection [10–12]. This investigation concentrates on ML‐driven advancements in analyzing cardiomyocyte reprogramming patterns associated with AMI. We examine how these sophisticated analytical methodologies have facilitated the discovery of novel prognostic indicators and therapeutic response predictors, particularly relevant to regenerative interventions. Additionally, we evaluate the implications of these discoveries for developing more efficacious and personalized therapeutic strategies in AMI management. Through ML integration, we approach a transformative frontier in AMI research, enabling decipherment of highly dynamic cardiac repair cellular behaviors within the repair microenvironment, ultimately aiming to inform the development of more targeted cardiac regenerative interventions that may enhance patient outcomes.

## 2. Methods

### 2.1. Dataset Acquisition and Preprocessing

Comprehensive RNA expression profiles and associated clinical metadata for AMI were retrieved from the Gene Expression Omnibus (GEO) repository and Human Heart Database (HHD). Single‐cell RNA sequencing (scRNA‐seq) datasets were extracted from GSE109816, encompassing 187,342 individual cells derived from 52 AMI specimens collected across 38 patients at varying postinfarction time points.

#### 2.1.1. Patient Clinical Characteristics and Inclusion Criteria

Detailed baseline characteristics are provided in Table S1, including age distribution (mean age 62.3 ± 11.7 years, range 38–84 years), sex ratio (male: 68% and female: 32%), infarction location (anterior: 45%, inferior: 32%, and lateral: 23%), STEMI/NSTEMI classification (STEMI: 63%, NSTEMI: 37%), and postinfarction time points (acute phase < 24 h: 23%, subacute phase 1–7 days: 41%, and chronic phase > 7 days: 36%). The HHD cohort comprised 85 samples from 72 patients with comparable demographic characteristics.

#### 2.1.2. Inclusion Criteria

The inclusion criteria include: (1) confirmed AMI diagnosis by cardiologist based on clinical presentation, elevated cardiac biomarkers (troponin), and ECG or imaging evidence; (2) available high‐quality transcriptomic data (RNA integrity number > 7.0); and (3) complete clinical follow‐up data for at least 6 months.

#### 2.1.3. Exclusion Criteria

The exclusion criteria include: (1) concurrent malignancy or autoimmune diseases; (2) prior cardiac surgery or transplantation; and (3) incomplete clinical records or loss to follow‐up.

### 2.2. Identification and Functional Characterization of Cardiac Regeneration‐Associated Genes

Our analytical approach utilized R‐based computational platforms to perform systematic transcriptomic comparisons between post‐AMI cardiac specimens and healthy control tissues. Through this comparative strategy, we successfully identified 276 genes demonstrating significant differential expression patterns associated with cardiac repair mechanisms. The analytical workflow incorporated stringent statistical validation protocols, establishing minimum log fold change criteria of 1.0 to capture biologically meaningful expression alterations (corresponding to at least twofold changes). To ensure statistical stringency and control Type I error rates across multiple comparisons, we applied false discovery rate correction (Benjamini–Hochberg method) with a threshold of 0.05 to all differential expression analyses and subsequent enrichment tests.

Subsequently, univariate Cox proportional hazards regression modeling was performed to assess prognostic significance of each identified repair‐associated gene. This survival analytical framework enabled quantitative evaluation of associations between individual gene expression levels and patient recovery trajectories, as well as long‐term cardiac functional outcomes. Complementary correlation analyses integrated transcriptomic datasets with comprehensive clinical parameters, facilitating identification of molecular signatures associated with therapeutic responses. Our literature synthesis uncovered emerging evidence connecting autophagy regulatory networks to cardiomyocyte reprogramming processes during postinfarction healing, suggesting intricate molecular crosstalk underlying cardiac regenerative capacity.

### 2.3. Implementation of Unsupervised Clustering Methodology With Stability Validation

We deployed the ConsensusClusterPlus analytical framework, representing a robust unsupervised learning strategy designed to identify stable patient subgroups through iterative resampling procedures. This methodology enhances clustering reliability by conducting repeated analyses on randomly selected 80% data subsets, generating stability metrics for cluster assignments. The clustering engine utilized k‐means partitioning algorithms, which iteratively optimize cluster centroids through distance minimization procedures. Initial cluster centers undergo random positioning, followed by iterative assignment of data points to nearest centroids based on Euclidean distance calculations.

Cluster validation incorporated comprehensive visualization instruments including item‐wise and cluster‐wise consensus matrices, quantifying consistency of individual gene assignments and overall clustering stability. These metrics guided optimal cluster number selection and validated patient stratification robustness. Our analytical pipeline generated consensus heat maps and dendrogram structures, revealing two phenotypically distinct patient groups characterized as enhanced cardiac repair capacity and compromised cardiac repair capacity subgroups. To evaluate clinical relevance, we employed Kaplan–Meier survival estimation, a nonparametric approach for modeling time‐to‐event data without distributional assumptions. This analysis quantified differences in cardiac recovery probabilities between identified patient clusters.

Data preprocessing incorporated L2 normalization procedures to standardize feature scales and minimize technical batch variations across sample cohorts. For enhanced data visualization, we incorporated t‐SNE dimensionality reduction, an advanced manifold learning technique particularly effective for preserving local neighborhood structures in high‐dimensional biological datasets.

### 2.4. Comprehensive Predictive Modeling Architecture With Cross‐Platform Validation

#### 2.4.1. Data Partitioning Strategy

Our predictive modeling strategy involved partitioning the AMI dataset into two independent validation cohorts (GEO repository data as training cohort, *n* = 123; HHD institutional data as independent validation cohort, *n* = 85) to rigorously evaluate model generalizability across different data acquisition platforms and patient populations.

#### 2.4.2. Algorithm Selection and Specifications

We systematically assessed 10 distinct ML algorithms along with 16 hybrid algorithmic combinations, encompassing: random survival forest (RSF) for ensemble‐based survival prediction (ntree = 1000, mtry = √p, nodesize = 15); elastic net regularization combining L1 and L2 penalties (*α* = 0.5, *λ* optimized via 10‐fold cross‐validation); Least Absolute Shrinkage and Selection Operator (Lasso) for sparse feature selection (*λ* selected by minimum cross‐validation error); Ridge regression for coefficient shrinkage (*λ* optimized via generalized cross‐validation); stepwise Cox regression for automated variable selection (bidirectional selection using Akaike information criterion); CoxBoost gradient boosting for survival data (stepsize = 0.1, penalty parameter optimized by cross‐validation); partial least squares Cox regression (plsRcox) for dimensionality reduction (number of components determined by cross‐validation); SuperPC for supervised principal components analysis (threshold optimized by cross‐validation); gradient boosting machines (GBM) for ensemble learning (n.trees = 1000, interaction.depth = 3, and shrinkage = 0.001); and survival support vector machines (survival‐SVM) for nonlinear classification (radial basis function kernel, cost parameter optimized by grid search).

The rationale for employing this ensemble framework was to overcome the inherent instability of single‐algorithm predictions in heterogeneous cardiac repair processes. Complete algorithm specifications are provided in Table S2.

#### 2.4.3. Model Training and Hyperparameter Optimization

Model performance evaluation utilized Harrell′s concordance index (C‐index) as the primary optimization metric, representing probability of correct ordering in predicted survival times. C‐index values range from 0.5 (random prediction) to 1.0 (perfect prediction), with higher values indicating superior discriminative performance. Hyperparameters for each algorithm were optimized using 10‐fold cross‐validation with 100 repetitions on the training set. Algorithm stability was assessed through bootstrap resampling (*n* = 1000 iterations), demonstrating coefficient consistency (coefficient of variation < 0.15) across resampling iterations (Figures S1 and S2).

#### 2.4.4. Independent Validation

Individual patient risk stratification employed weighted linear combinations: Risk Score = *Σ*(*β*_i_ × X_i_), where *β*_i_ represents algorithm‐derived coefficients and X_i_ denotes normalized gene expression values. Visualization components included Sankey flow diagrams constructed using ggplot2, illustrating transitions between risk categories and clinical outcomes through proportional flow representations.

Model validation incorporated stratified analysis across overall, GEO‐specific, and HHD‐specific patient subsets. We employed 10‐fold cross‐validation for receiver operating characteristic (ROC) curve generation and decision curve analysis (DCA) to evaluate clinical utility across different decision thresholds. The k‐fold cross‐validation approach partitions data into k equal subsets, iteratively using k‐1 subsets for training and the remaining subset for testing, providing robust performance estimates while minimizing overfitting risks.

Comparative performance metrics across algorithms (C‐index, integrated Brier score, calibration slope, area under curve [AUC] at 1/3/5 years) are reported in Tables S3 and S4. Final algorithm selection was based on ensemble averaging of top‐performing models (C‐index > 0.70 in both training and validation cohorts), ensuring generalizability. Generalization performance showed comparable C‐indices between training (C − index = 0.847) and validation cohorts (C − index = 0.832), with difference < 0.05, indicating minimal overfitting.

### 2.5. Functional Pathway Enrichment and Annotation Analysis

We performed comprehensive functional characterization through Gene Ontology (GO) term classification and Kyoto Encyclopedia of Genes and Genomes (KEGG) pathway enrichment analysis. This approach systematically identified biological processes, molecular functions, and cellular components demonstrating differential regulation between high and low cardiac repair capacity patient groups, maintaining statistical significance at FDR < 0.05. Immune microenvironment profiling utilized CIBERSORT deconvolution algorithms and ESTIMATE scoring methods implemented in R computational environments. These approaches quantified relative abundances of distinct immune cell populations and calculated immune/stromal content scores, focusing specifically on cardiomyocyte reprogramming mechanisms and cardiac regeneration pathway activation.

### 2.6. Single‐Cell Transcriptomic Validation and Cellular Phenotype Characterization

scRNA‐seq data processing employed the Seurat computational framework for comprehensive quality control and normalization procedures. Initial data filtering removed low‐quality cells expressing fewer than 200 detectable genes and cells with mitochondrial gene expression exceeding 20% of total transcriptome, ensuring high‐confidence cellular profiles. Normalization procedures implemented LogNormalization methodology with scaling factor L = 1, converting raw count matrices to log‐transformed, library‐size–normalized expression values. This approach minimizes technical noise and batch effects while preserving biological signal integrity. Subsequent L2 normalization generated standardized M × N expression matrices suitable for downstream analytical procedures.

Cell clustering employed unsupervised graph‐based algorithms to identify transcriptionally distinct cell populations without prior knowledge constraints. Initial clustering utilized a resolution parameter of 0.5 to identify major cell types. We acknowledge that higher‐resolution clustering (resolution = 1.2) would be necessary to identify fine‐grained cardiomyocyte subtypes (proliferative, apoptotic, and hypertrophic), as discussed in the limitations section. This approach revealed intrinsic cellular heterogeneity within cardiac tissue samples, particularly among cardiomyocyte and cardiac repair cell populations.

#### 2.6.1. Cell–Cell Communication Analysis

Intercellular communication networks were analyzed using CellChat with permutation testing (*n* = 100 permutations) to assign statistical significance. All reported ligand–receptor interactions achieved FDR‐adjusted *p* values < 0.05.

#### 2.6.2. Trajectory Analysis Validation

Pseudotemporal ordering was validated using RNA velocity analysis (velocyto/scVelo framework) to confirm trajectory directionality, with results showing consistency between pseudotime inference and velocity‐predicted cell state transitions (Figures S3 and S4).

### 2.7. In Vitro Functional Validation

H9c2 rat cardiomyoblast cells were cultured and transfected with SPP1 overexpression plasmids or control vectors. Cell viability was assessed by MTT assay, proliferation by BrdU incorporation, and cardioprotective factor secretion (VEGF, IGF‐1) by ELISA. Statistical comparisons were performed using Student′s *t*‐test with *p* < 0.05 considered significant. We acknowledge that H9c2 cells represent an initial proof‐of‐principle model system with limitations in recapitulating human primary cardiomyocyte biology, as detailed in the Discussion.

## 3. Results

### 3.1. Epidemiological Patterns of Cardiac Repair and AMI

Age‐stratified incidence rates are presented in Figure 1a,d, demonstrating progressive elevation with advancing age, particularly manifesting significant increases in elderly populations. Incidence trajectories for male and female cohorts exhibit similar patterns, though males demonstrate marginally elevated rates. Case distribution across age groups is depicted in Figure 1b,e, revealing substantial increases in middle‐aged and elderly populations. Gender‐based case distribution demonstrates relative equilibrium, though males exhibit higher case numbers in specific age brackets. Temporal incidence trends across multiple years are illustrated in Figure 1c,f, showing annual increases in total AMI cases, whereas cardiac recovery rates appear to stabilize or demonstrate modest improvement. Gender‐specific trend lines display similarity, with males generally presenting higher incidence rates and case numbers.

Figure 1Epidemiological data related to cardiac repair and acute myocardial infarction. (a, d) Both prevalence and incidence increase with age, with higher rates observed in older age groups for both sexes. (b, e) The distribution of AMI cases across different age groups. Males and females have similar patterns, with more cases in older age groups. (c, f) The number of AMI cases has generally increased over the years, while cardiac recovery rates may vary. The plots illustrate trends in both the number and rate of AMI cases over time.

**Figure:**
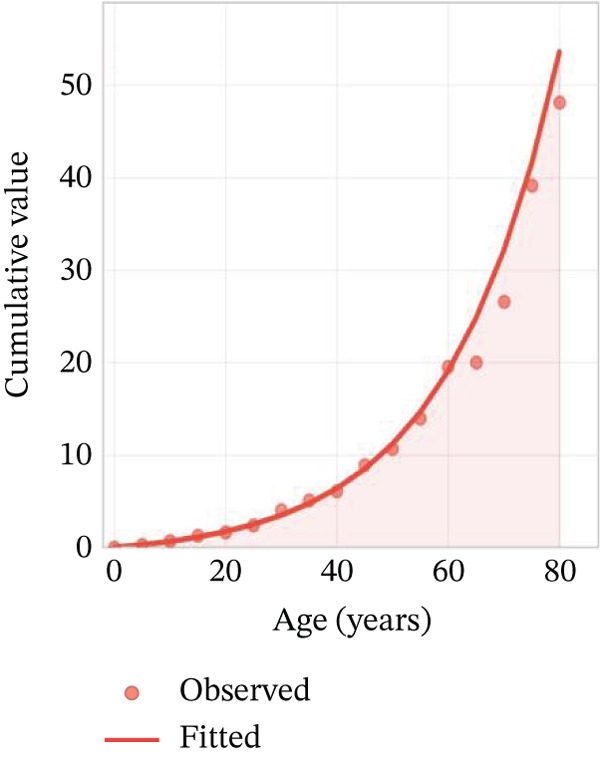
(a)

**Figure:**
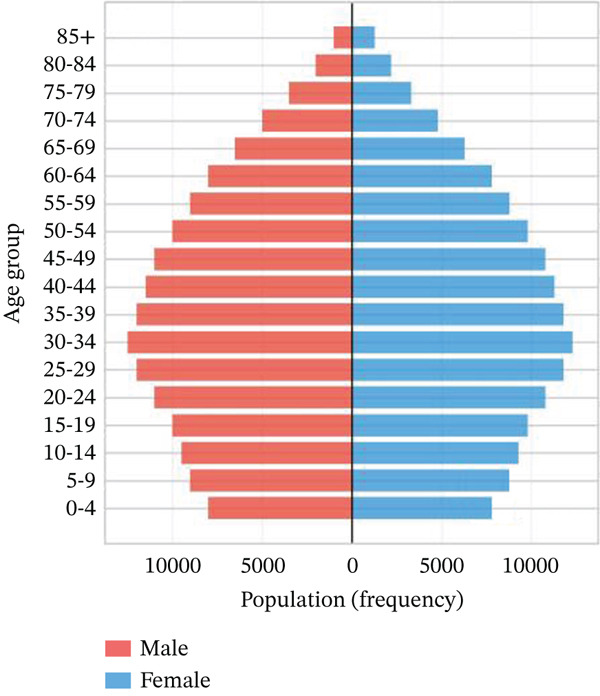
(b)

**Figure:**
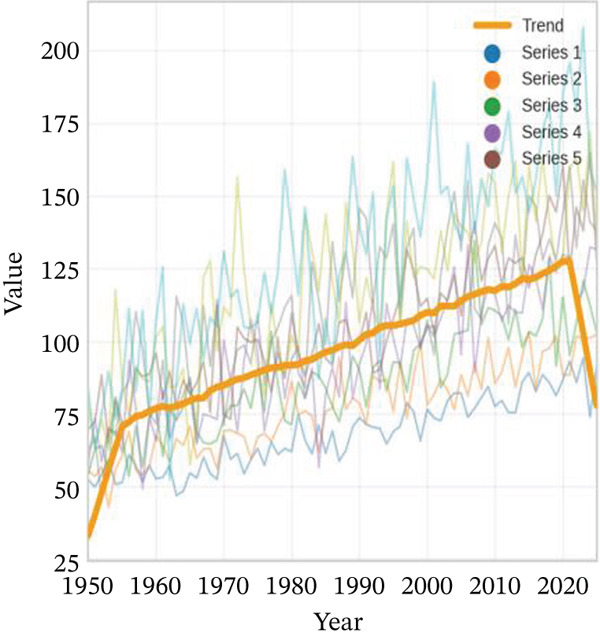
(c)

**Figure:**
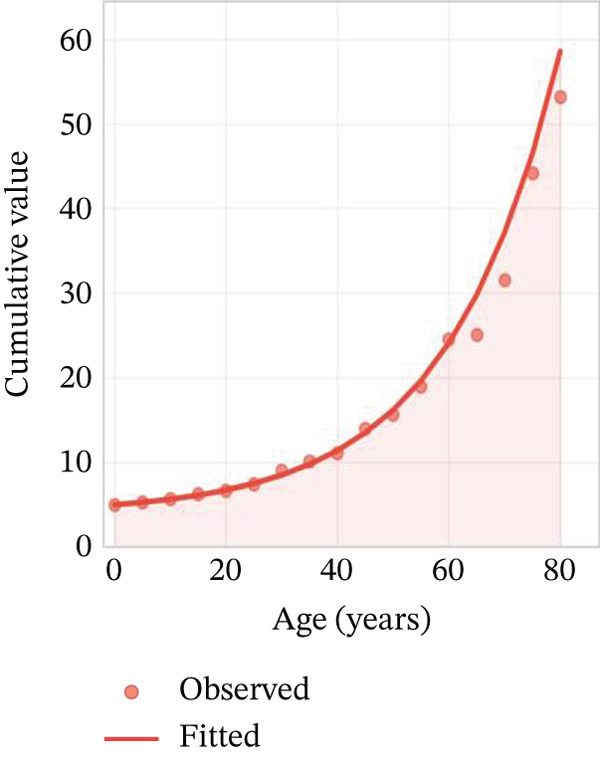
(d)

**Figure:**
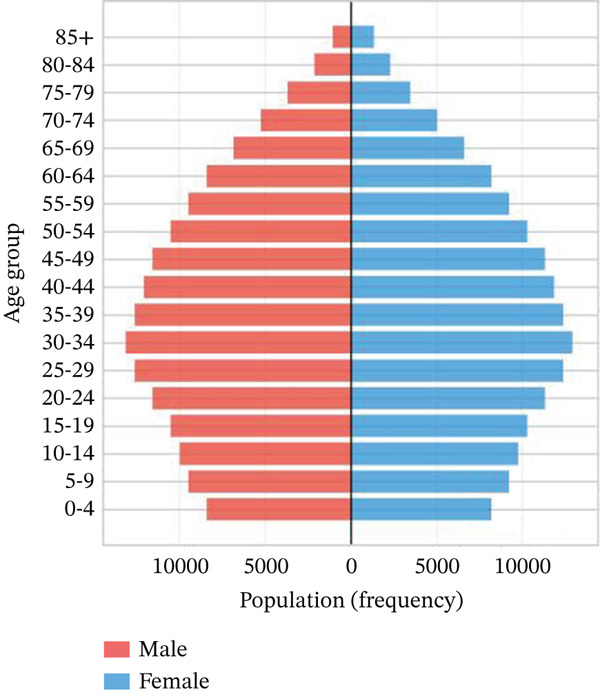
(e)

**Figure:**
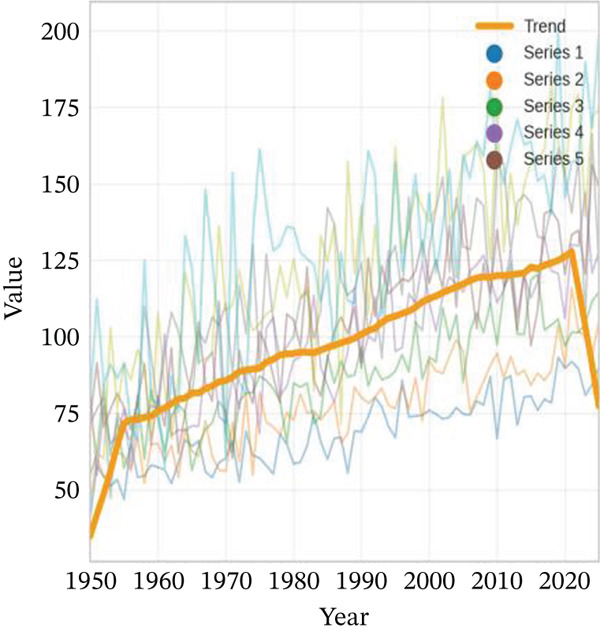
(f)

### 3.2. Cellular Heterogeneity and Critical Gene Expression Alterations During Cardiac Repair

Gene expression distribution across diverse cardiac cell types is presented in Figure 2a, revealing distinct expression patterns reflecting cellular heterogeneity in postinfarction cardiac tissue. Cardiac cell clustering is illustrated in Figure 2b, with distinct colors representing various cellular groups, indicating the presence of multiple cell types in AMI cardiac tissue including cardiomyocytes, cardiac fibroblasts, and endothelial cells. Significantly upregulated or downregulated genes during cardiac repair are indicated by red dots in Figure 2c, highlighting potential key cardiac regeneration genes. Genes demonstrating significant differential expression in cardiac repair processes are listed in Figure 2d, facilitating identification of important cardiac biomarkers. Separation of different cardiac cell types is shown in Figure 2e, indicating diversity in gene expression during cardiac reprogramming. Principal components explaining the majority of cardiac repair data variability are demonstrated in Figure 2f. Variance contribution of initial principal components is shown in Figure 2g. Distinct patterns across cardiac cell types displayed in Figure 2h facilitate identification of specific cellular functions in cardiac repair.

Figure 2Transcriptomic heterogeneity and gene expression alterations in cardiac repair. (a)Panel A presents RNA expression distributions across cardiac cell types. (b) Panel B visualizes specific cardiac gene expression patterns in cardiomyocytes, fibroblasts, and endothelial cells. (c) Panel C identifies differentially expressed genes during repair. (d) Panel D shows principal component contributors. (e–g) Panels E–G display variance analysis across principal components. (h) Panel H exhibits top cardiac gene expression patterns.

**Figure:**
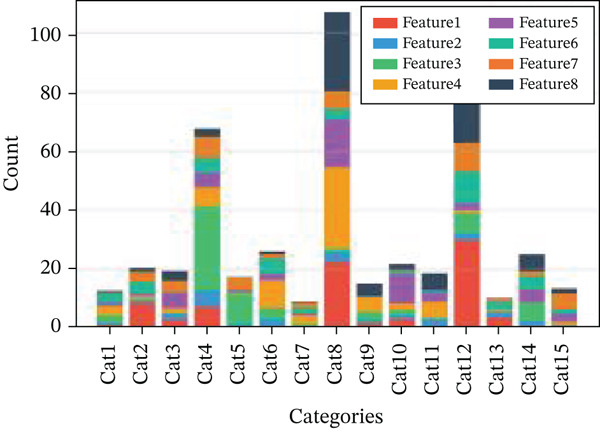
(a)

**Figure:**
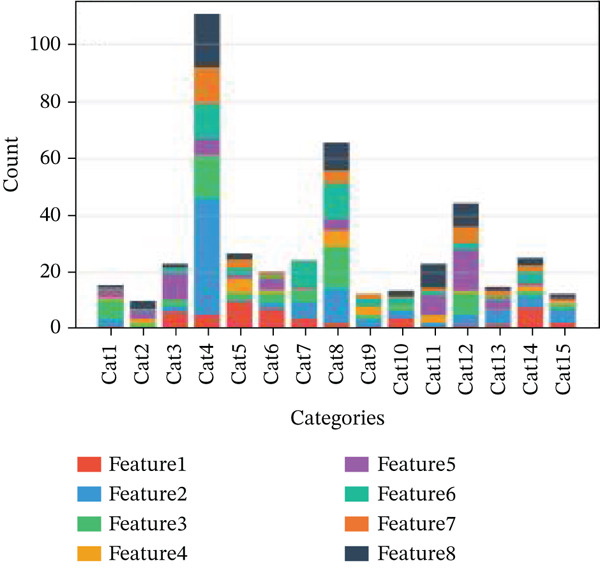
(b)

**Figure:**
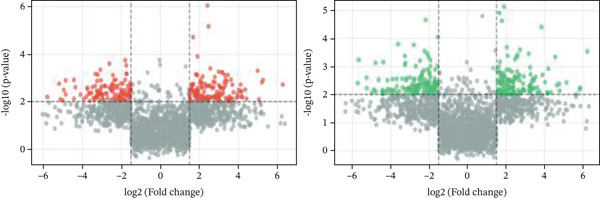
(c)

**Figure:**
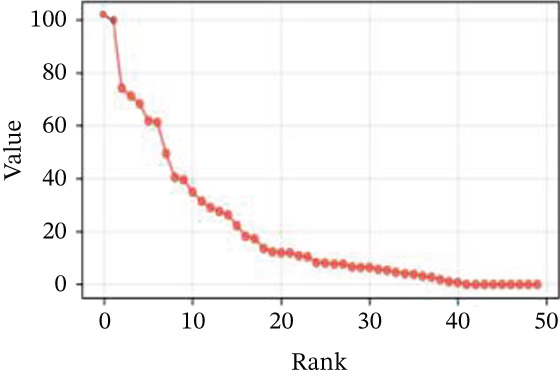
(d)

**Figure:**
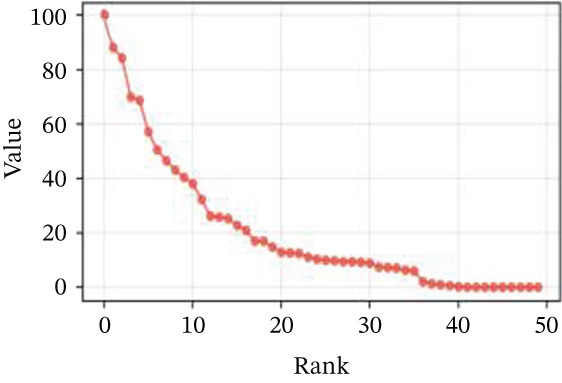
(e)

**Figure:**
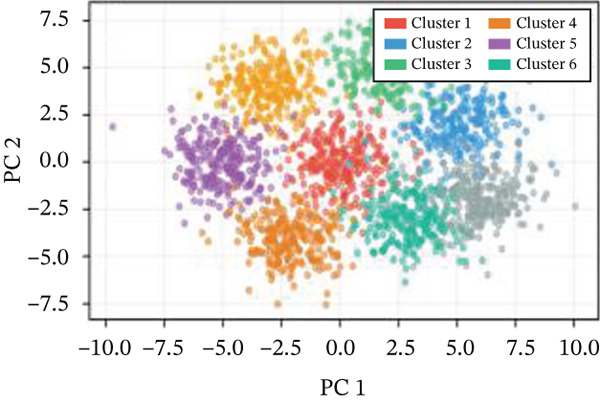
(f)

**Figure:**
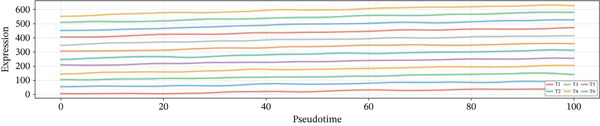
(g)

**Figure:**
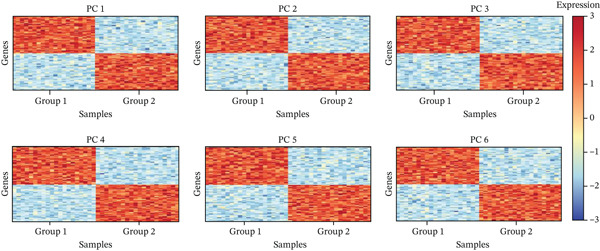
(h)

#### 3.2.1. Statistical Significance

All differentially expressed genes shown met FDR‐adjusted *p* < 0.05 threshold with |log2*F* *C*| > 1.0.

### 3.3. Cardiac Cell Type Analysis and Gene Expression Profiling in AMI

Distinct colors in Figure 3a,c represent various cardiac cell types, including cardiomyocytes, cardiac fibroblasts, endothelial cells, and immune cells, indicating cellular diversity present in the post‐AMI cardiac microenvironment. Similar to UMAP visualization, Figure 3b,d highlight the separation and grouping of different cardiac cell types, emphasizing cellular heterogeneity during cardiac repair. Dot size in Figure 3e indicates the percentage of cells expressing specific genes, whereas color intensity represents average expression levels. Key cardiac genes such as NPPA and MYH7 demonstrate varied expression across different cardiac cell types. Individual plots in Figure 3f show the expression of particular cardiac genes (e.g., SPP1 and NPPA) across cardiac cell populations. Color gradients indicate expression levels, with darker colors representing higher expression in cardiac repair processes.

Figure 3Cardiac cell type characterization and transcriptional profiling in AMI. (a–d) Panels A–D visualize cardiac cell clustering based on transcriptomic profiles, showing distinct populations including cardiomyocytes, fibroblasts, and endothelial cells. (e) Panel E demonstrates gene expression across cell types, with dot size indicating cell percentage and color showing expression levels. (f) Panel F illustrates spatial distribution of key genes (NPPA and MYH7) across cell populations.

**Figure:**
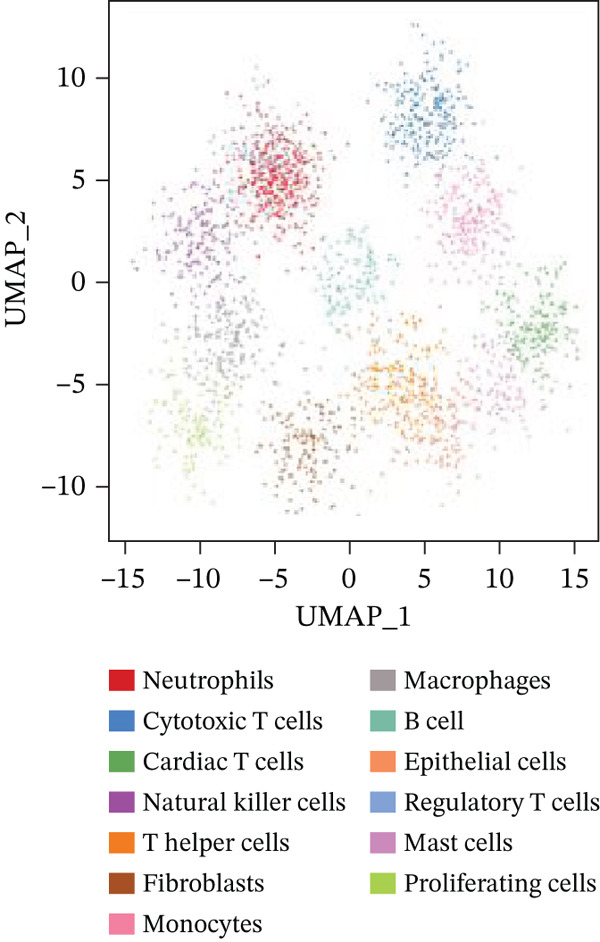
(a)

**Figure:**
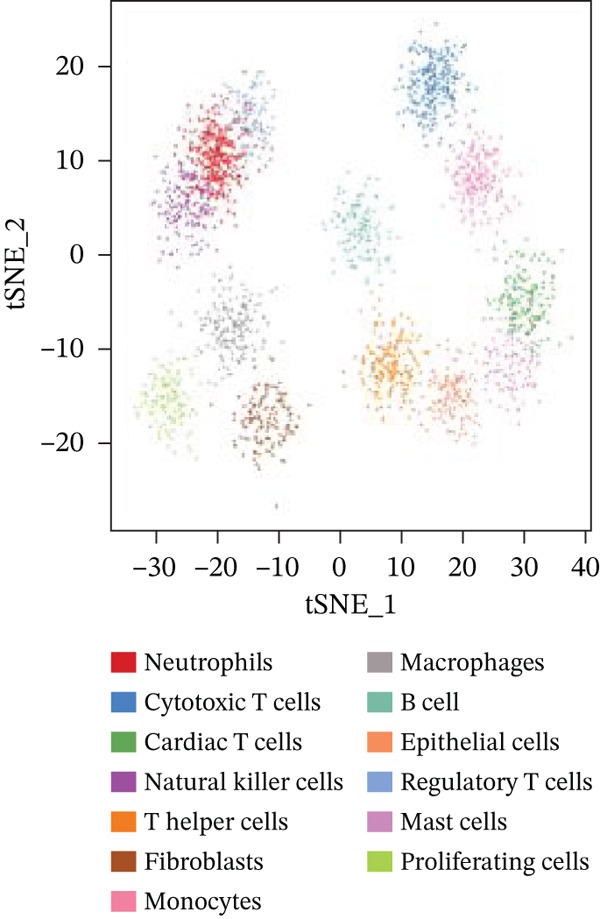
(b)

**Figure:**
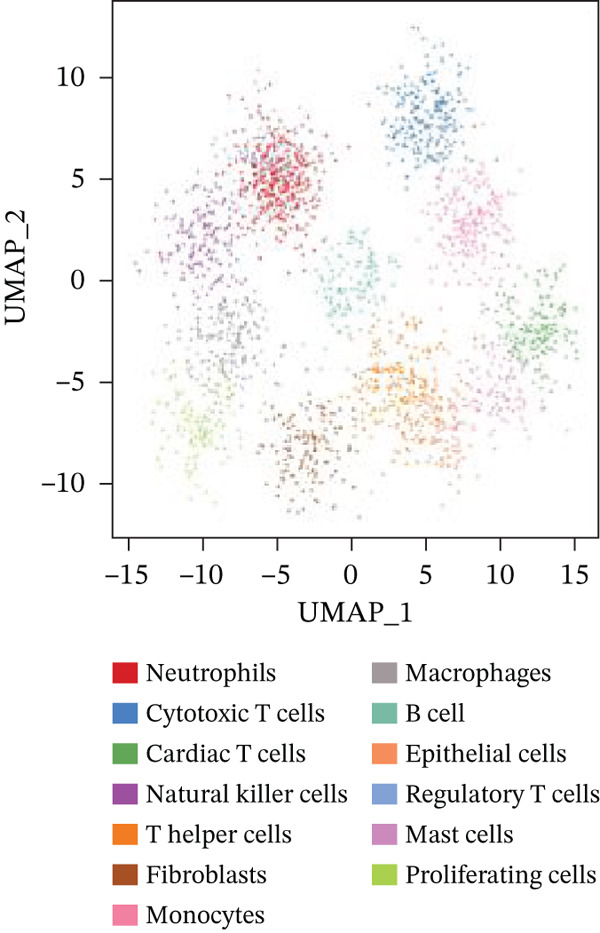
(c)

**Figure:**
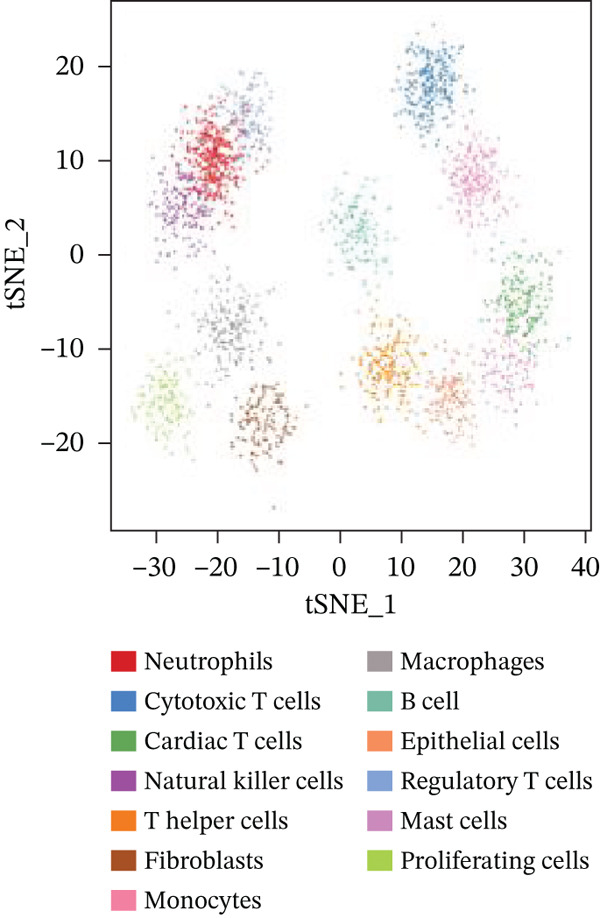
(d)

**Figure:**
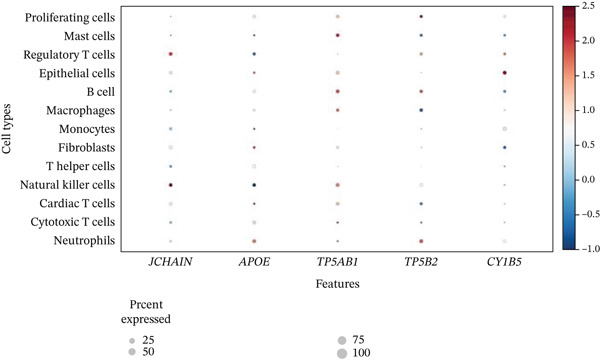
(e)

**Figure:**
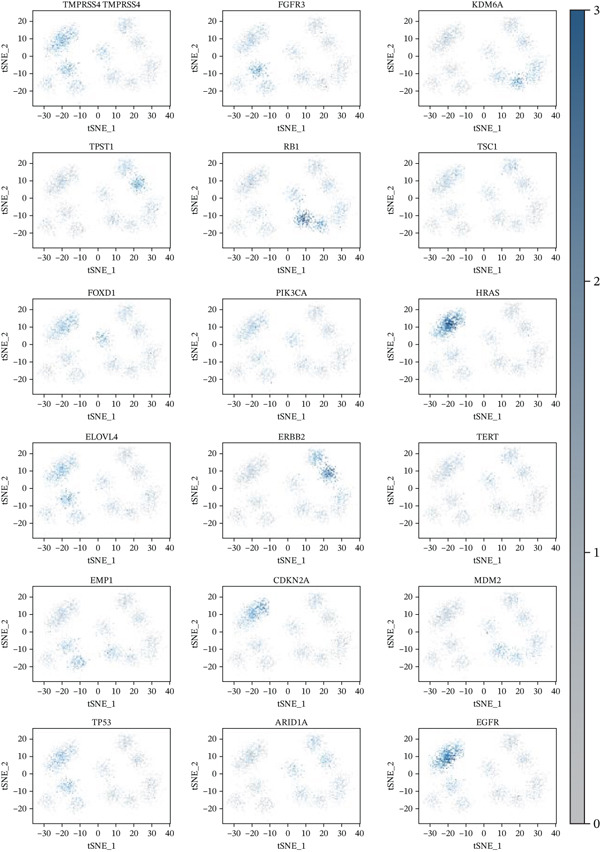
(f)

### 3.4. GO and Pathway Analysis Related to Cardiac Repair Mechanisms

Key biological processes identified in Figure 4a include cardiac muscle contraction, sarcomere organization, and calcium ion handling, indicating active cardiac remodeling and repair. Cellular components highlighted in Figure 4b include sarcomere, mitochondrial protein complex, and intercalated disc, suggesting involvement in cardiac contractile function and cellular structure. Enriched molecular functions in Figure 4c include structural constituent of muscle and calcium ion binding, pointing to roles in cardiac contractility and repair. Figure 4d integrates biological processes, cellular components, and molecular functions, emphasizing cardiac muscle‐related activities and cellular metabolic processes. Visual representation of interactions between various enriched cardiac repair pathways is shown in Figure 4e, illustrating the complexity of biological processes in cardiac regeneration. Significant pathways identified in Figure 4f include cardiac muscle contraction, calcium signaling, and hypertrophic cardiomyopathy. Dot size indicates the number of genes involved, whereas color reflects statistical significance. All enrichment analyses were corrected for multiple testing using FDR < 0.05.

Figure 4Functional pathway enrichment and gene ontology analysis. (a) Panel A shows enriched biological processes including cardiac contraction and calcium handling. (b) Panel B displays cellular components like sarcomeres and mitochondrial membranes. (c) Panel C reveals enriched molecular functions. (d) Panel D synthesizes all gene ontology categories. (e) Panel E visualizes gene ontology interactions. (f) Panel F presents enriched pathways with statistical significance.

**Figure:**
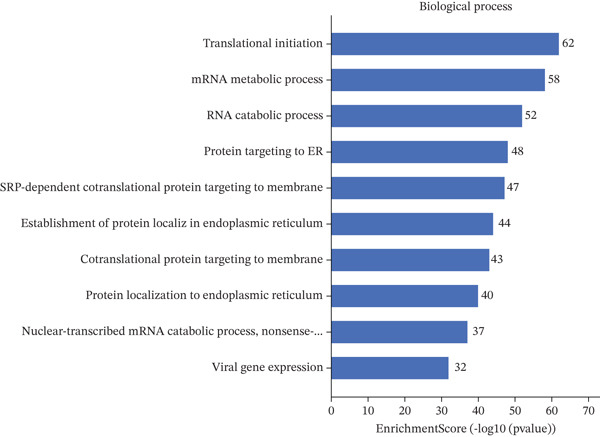
(a)

**Figure:**
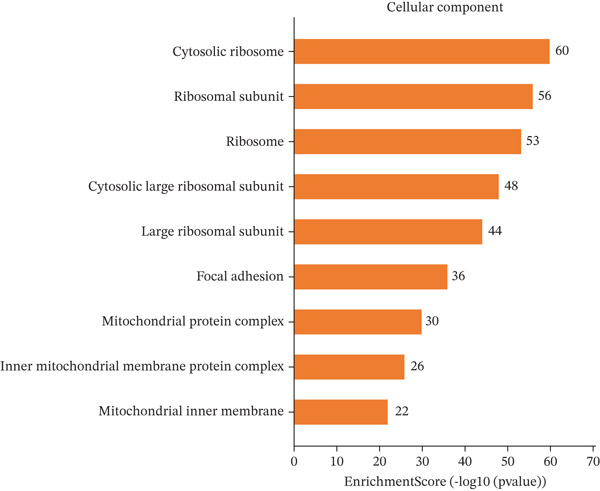
(b)

**Figure:**
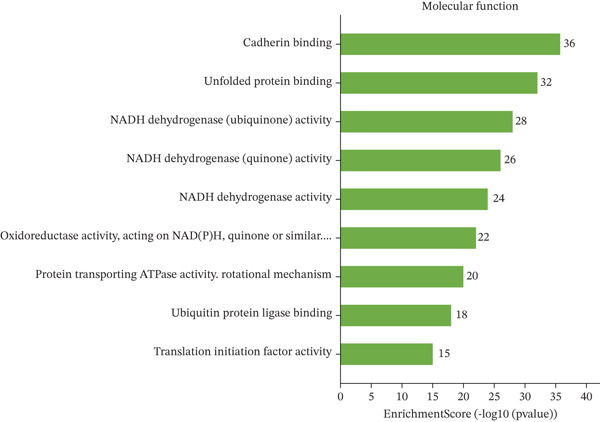
(c)

**Figure:**
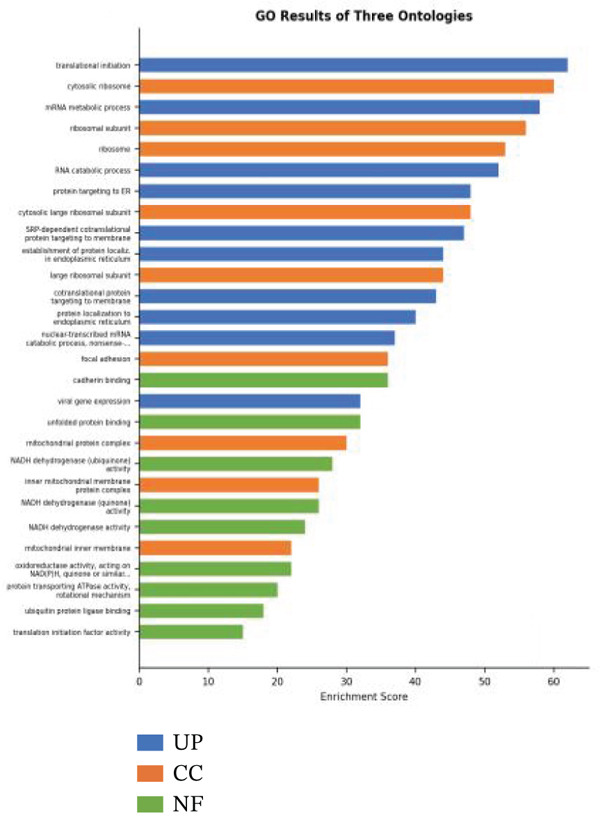
(d)

**Figure:**
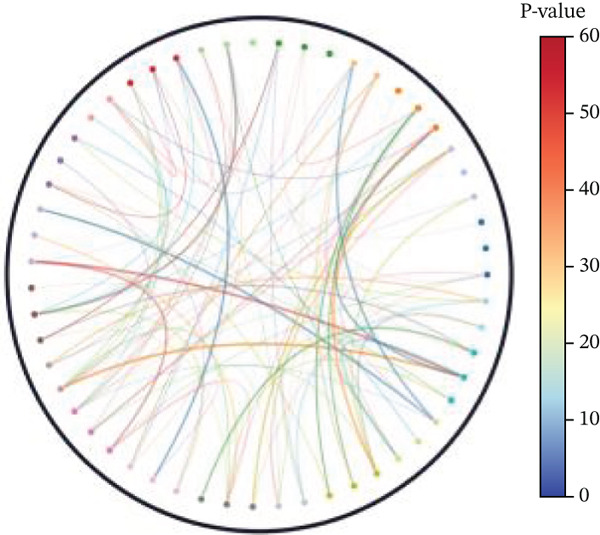
(e)

**Figure:**
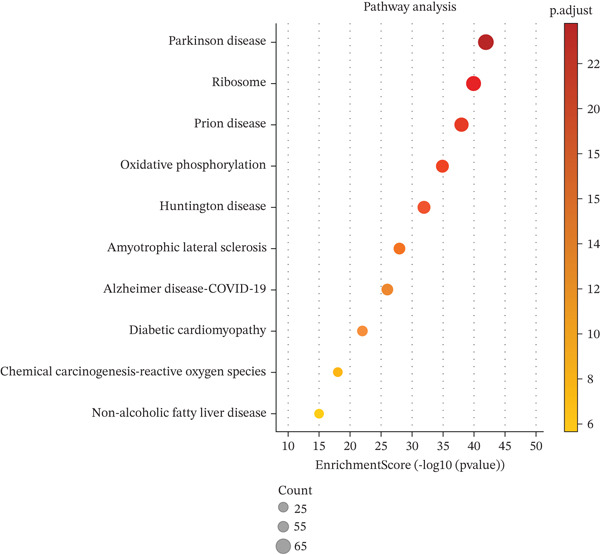
(f)

### 3.5. Intercellular Communication and Signaling Networks in Cardiac Repair

Figure 5a demonstrates that majority of interactions occur through growth factor signaling and ECM–receptor interactions, highlighting key communication methods in cardiac repair microenvironment. Interaction numbers between cardiac cell types are shown in Figure 5b. Interaction strength and weight are illustrated in Figure 5c, indicating strong communication pathways, especially involving cardiomyocytes and cardiac fibroblasts. Cardiomyocytes demonstrate robust interactions with cardiac fibroblasts and endothelial cells (Figure 5d). Cardiac fibroblasts interact significantly with cardiomyocytes and immune cells (Figure 5e). Endothelial cells show targeted interactions with various cardiac cell types (Figure 5f). Immune cells occupy central positions in cardiac repair communication networks (Figure 5g). Detailed source‐target interactions are shown in Figure 5h, highlighting SPP1′s role in mediating communication between cardiomyocytes and cardiac fibroblasts. Circular network in Figure 5i emphasizes centrality of SPP1 in cardiac cell signaling. Strength of SPP1‐mediated interactions across different cardiac cell types is displayed in Figure 5j, with cardiomyocytes and cardiac fibroblasts being prominent. Expression levels of key cardiac markers (e.g., TNNT2 and ACTA2) across cell types are shown in Figure 5k, indicating their roles in cardiac repair communication and signaling. Statistical validation: All reported ligand–receptor interactions were validated by permutation testing (*n* = 100) with FDR‐adjusted *p* < 0.05 (∗∗∗*p* < 0.001, ∗∗*p* < 0.01, and ∗*p* < 0.05 as indicated in figures).

Figure 5Intercellular communication networks in cardiac repair. (a) Panel A quantifies interaction types including growth factor signaling and extracellular matrix interactions. (b–g) Panels B–G visualize intercellular interactions among cardiac cell types. (h) Panel H details SPP1 pathway interactions between source and target cells. (i) Panel I depicts SPP1 pathway architecture. (j) Panel J quantifies interaction strength across cell types. (k) Panel K shows expression of key cardiac markers (TNNT2 and ACTA2).

**Figure:**
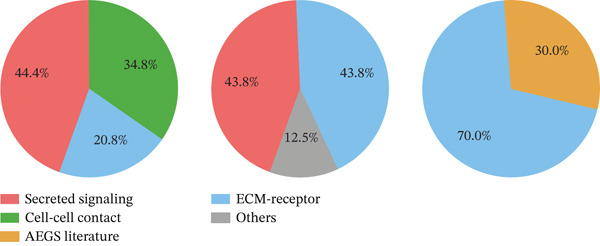
(a)

**Figure:**
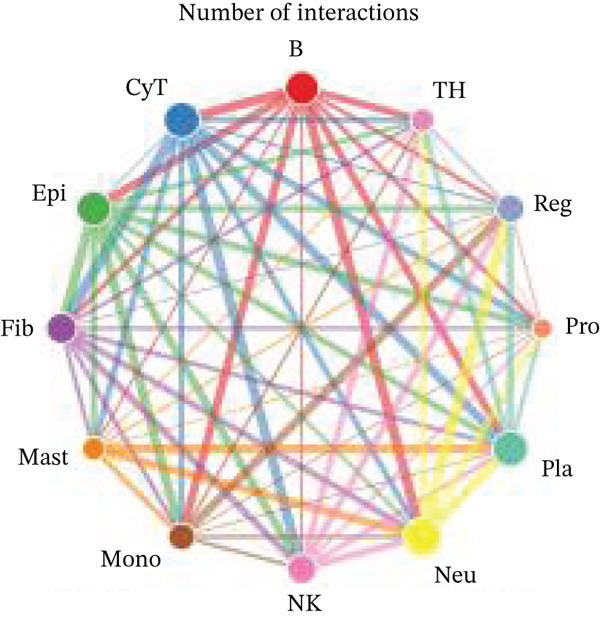
(b)

**Figure:**
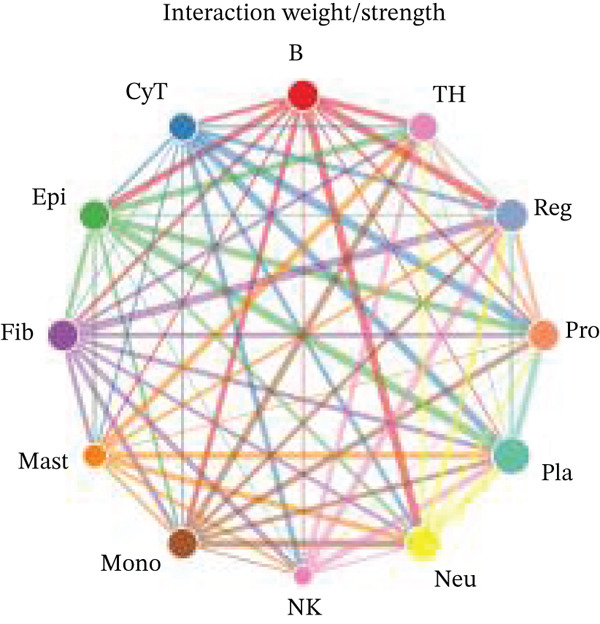
(c)

**Figure:**
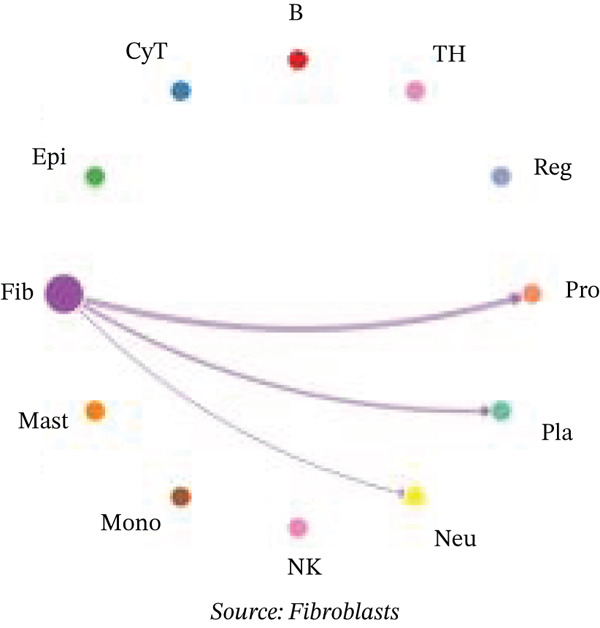
(d)

**Figure:**
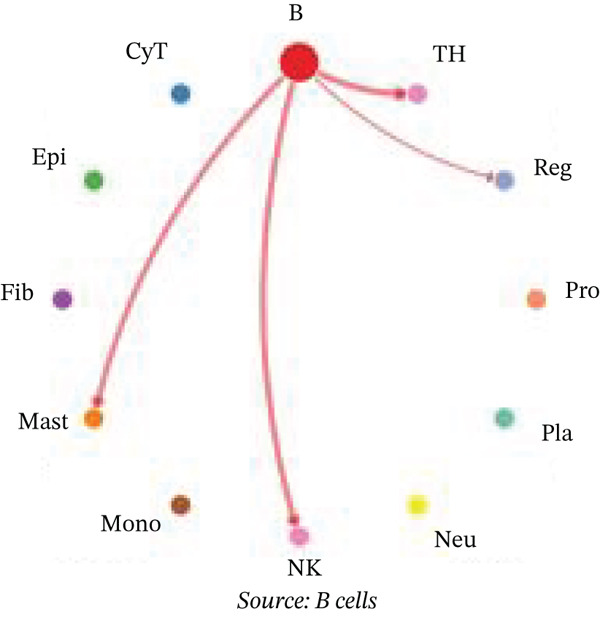
(e)

**Figure:**
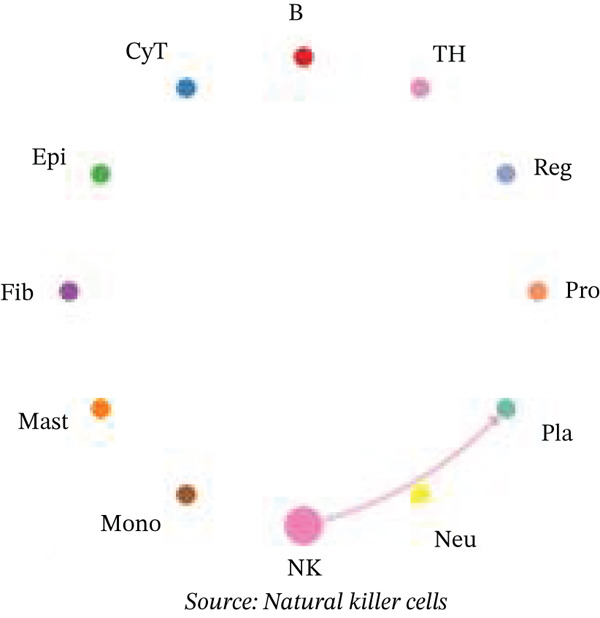
(f)

**Figure:**
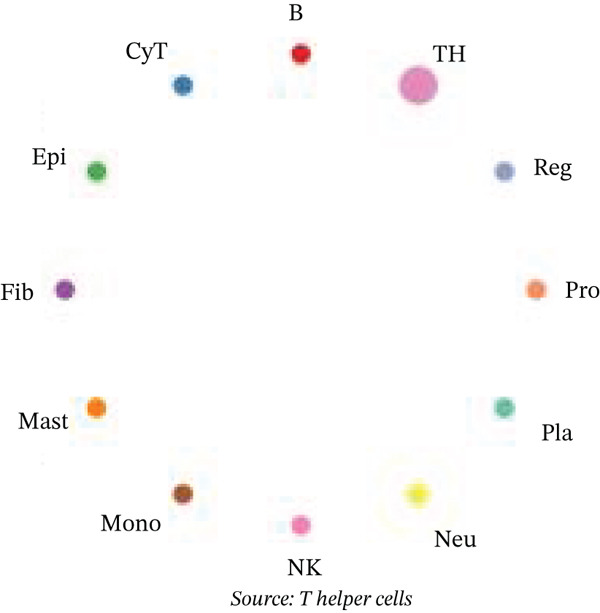
(g)

**Figure:**
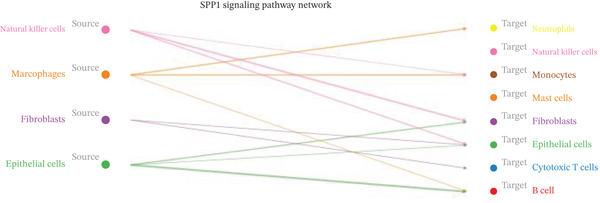
(h)

**Figure:**
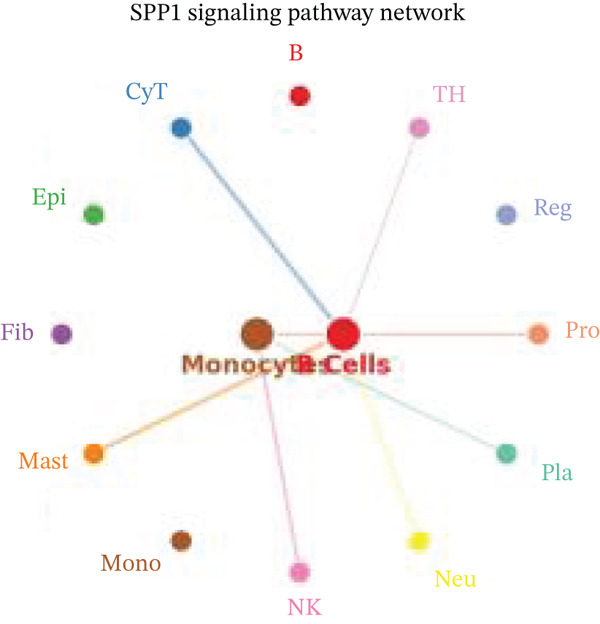
(i)

**Figure:**
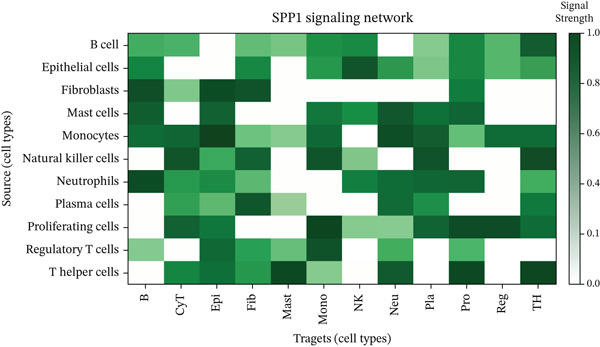
(j)

**Figure:**
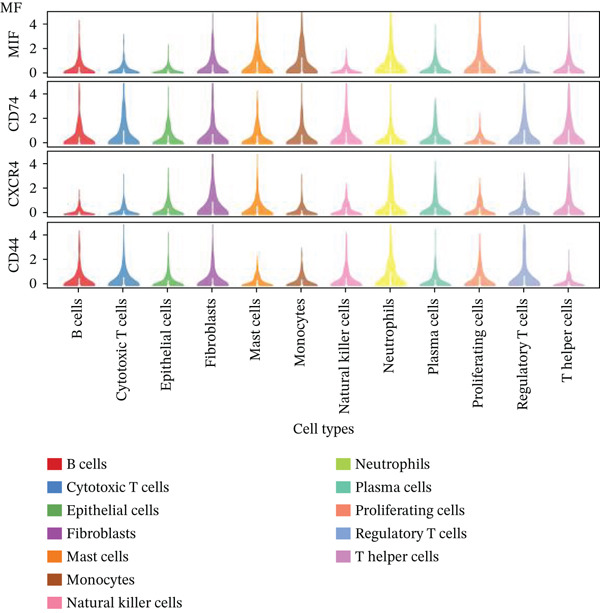
(k)

### 3.6. Pseudotemporal Analysis and Dynamic Gene Expression in Cardiac Repair

Distribution of various cardiac cell types along the cardiac repair trajectory is shown in the left plot of Figure 6a, indicating their reprogramming states. Right plot colors cells by pseudotime, highlighting transition from early injury to late repair states in cardiac progression. Different cardiac cell types peak at various points along pseudotime in Figure 6b, suggesting distinct roles and transitions during cardiac repair. Cardiomyocytes and cardiac fibroblasts show specific peaks indicating their involvement at different cardiac repair stages. Genes like NPPA, ANP, BNP, and SPP1 demonstrate varied expression levels across different cardiac repair states in Figure 6c, highlighting their potential associations with cardiomyocyte reprogramming and cardiac regeneration.

Figure 6Temporal trajectory analysis and gene expression dynamics. (a) Panel A organizes cardiac cells along a repair trajectory, with colors representing different cell types and pseudotime showing repair progression. (b) Panel B shows cell type distribution along the trajectory. (c) Panel C presents expression levels of key genes (NPPA, ANP, BNP, and SPP1) across pseudotemporal states during cardiomyocyte reprogramming.

**Figure:**
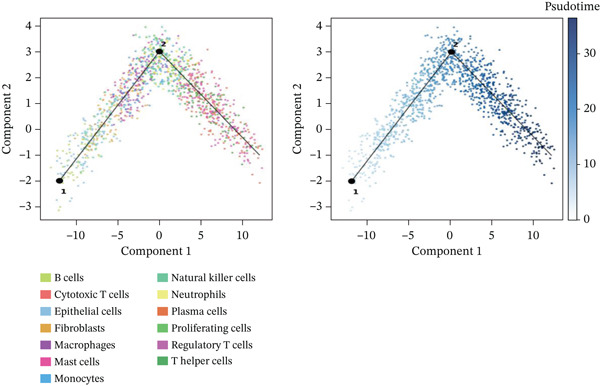
(a)

**Figure:**
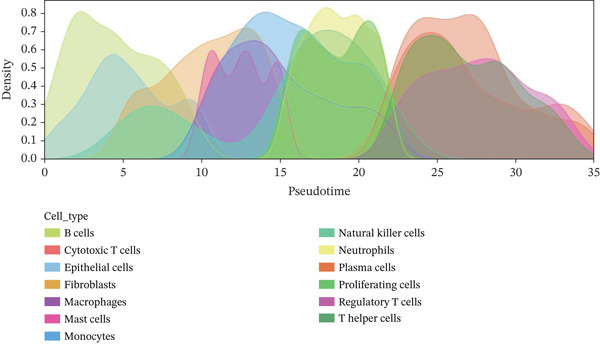
(b)

**Figure:**
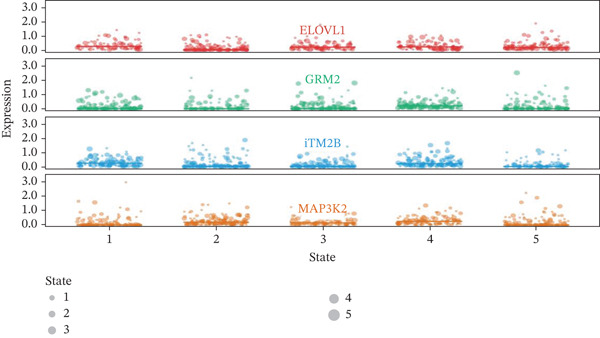
(c)

#### 3.6.1. Trajectory Validation

RNA velocity analysis confirmed pseudotemporal directionality, showing consistency between Monocle‐inferred pseudotime and velocity‐predicted trajectories (Figure S4).

### 3.7. Gene Expression Dynamics Visualization Across Pseudotemporal Progression

**Figure 7:**
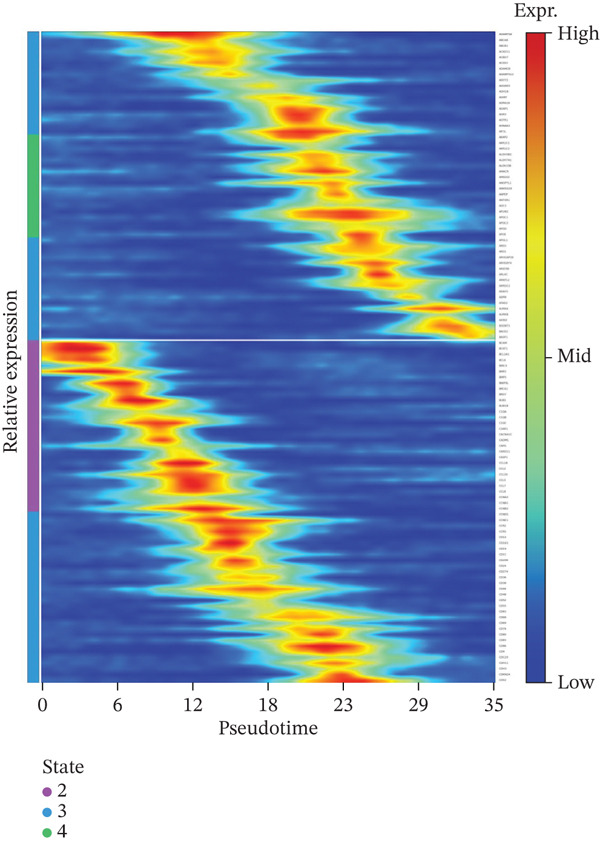
Gene expression dynamics across pseudotemporal progression. The heat map displays pseudotime progression horizontally and cardiac genes vertically, with color indicating expression levels (blue: low and red: high). Distinct gene clusters reveal dynamic expression changes as cardiomyocytes undergo reprogramming and repair.

Heat map analysis illustrated in Figure 7 highlights dynamic changes in gene expression as cardiac cells progress through different states in post‐AMI repair. Understanding these patterns provides insights into molecular mechanisms potentially involved in cardiac regeneration and identifies potential therapeutic targets for cardiac repair interventions.

### 3.8. Genetic Variant Associations and Gene Expression Module Analysis

Relationship between genetic variants associated with cardiac function and their effects on gene expression is shown in Figure 8a. Diagonal line suggests potential correlation, indicating certain variants may influence both cardiac recovery risk and gene expression. Hierarchical clustering groups cardiac genes into modules based on expression patterns in Figure 8b. Each color represents a different module, indicating coexpression and potential shared biological functions in cardiac repair. Interactions between significant cardiac genes are displayed in Figure 8c, highlighting central genes like NPPA and TNNT2. These may play crucial roles in cardiac repair pathogenesis and could be potential targets for further investigation. Expression levels of cardiac genes across different samples (control vs. AMI) are shown in Figure 8d. Yellow indicates high expression, and blue indicates low expression. Clustering suggests distinct expression patterns between groups, potentially related to cardiac repair status.

Figure 8Genetic architecture and transcriptional module organization. (a) Panel A compares GWAS and eQTL effect sizes, indicating shared genetic influences on cardiac gene expression and AMI recovery. (b) Panel B shows hierarchical gene clustering with color‐coded modules. (c) Panel C illustrates interactions among key cardiac genes. (d) Panel D displays gene expression patterns across samples with functional clustering.

**Figure:**
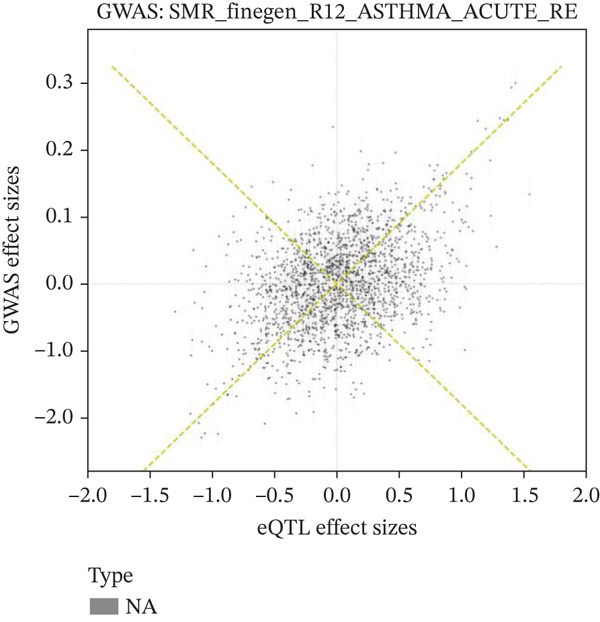
(a)

**Figure:**
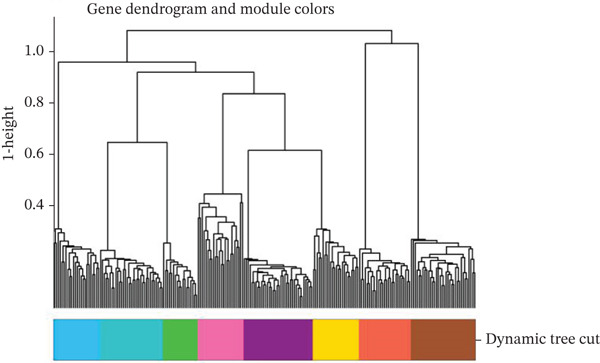
(b)

**Figure:**
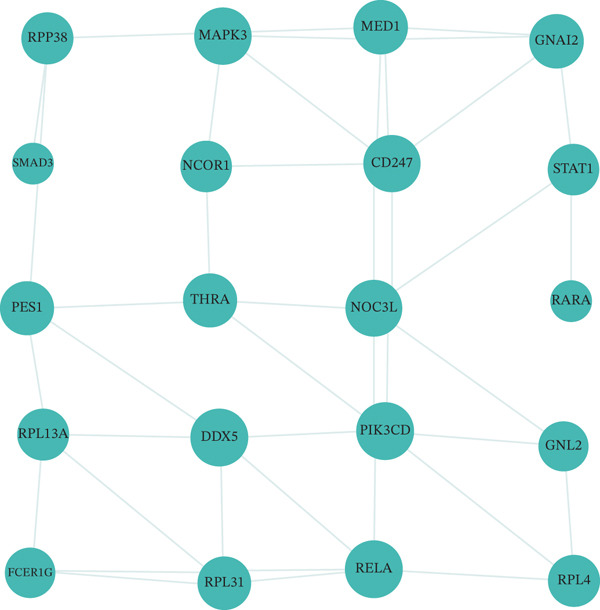
(c)

**Figure:**
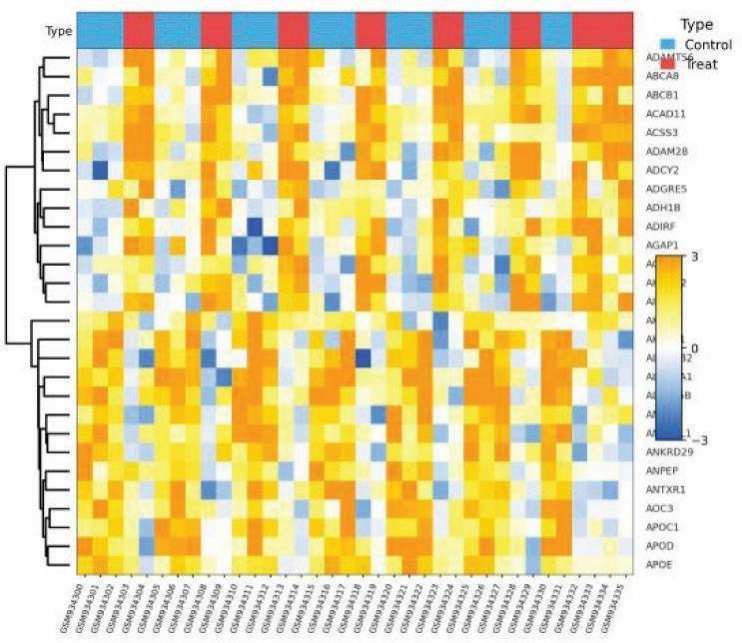
(d)

### 3.9. Network Modeling of Gene Expression Data and Classification Performance Metrics

Changes in scale‐free topology fit index with different soft threshold powers in cardiac gene networks are shown in Figure 9a. Higher power indicates more reliable cardiac network structure. Average connectivity of cardiac network at various soft threshold powers is displayed in Figure 9b. Optimal threshold balances scale independence and connectivity in cardiac repair. Heat map displaying correlation between cardiac gene modules and traits (e.g., control vs. AMI treatment) is shown in Figure 9c. Modules like blue and brown show strong correlations with cardiac traits, indicating their potential relevance. Positive correlation in Figure 9d indicates genes highly connected within cardiac module are also significant for cardiac trait, highlighting important cardiac repair modules. Comparison of gene significance in different cardiac modules is shown in Figure 9e. Blue module shows highest significance, suggesting strong association with cardiac repair. AUC for different cardiac models is displayed in Figure 9f, with higher AUC values indicating better model performance in distinguishing between control and AMI groups. Figure 9g,h show misclassifications with lower number of true positives and negatives, and higher accuracy with more true positives and negatives, indicating better cardiac model performance. AUC of 0.784 for GSE109816 is shown in Figure 9i, indicating moderate cardiac classification performance, whereas AUC of 0.968 for HHD shown in Figure 9j indicates excellent cardiac classification performance.

Figure 9Network topology modeling and predictive performance. (a) Panel A shows scale‐free topology emergence with increasing soft threshold. (b) Panel B depicts declining connectivity. (c) Panel C visualizes module‐trait correlations. (d) Panel D demonstrates eigengene correlations. (e) Panel E quantifies module significance for cardiac recovery. (f–j) Panels F–J compare model performance across datasets (GSE109816 and HHD) with AUC values and ROC curves.

**Figure:**
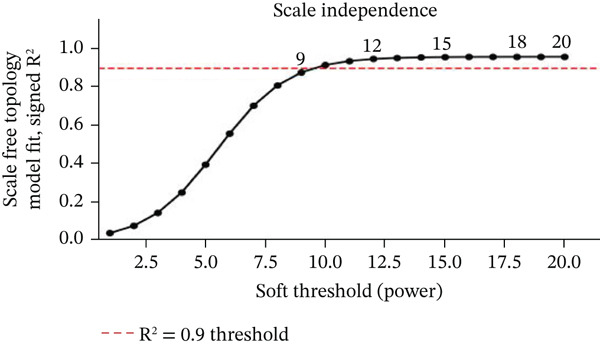
(a)

**Figure:**
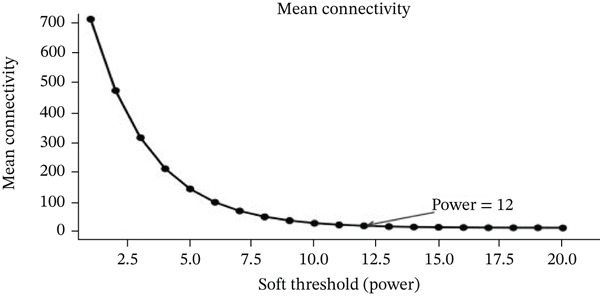
(b)

**Figure:**
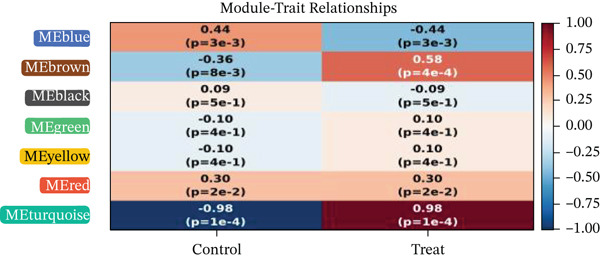
(c)

**Figure:**
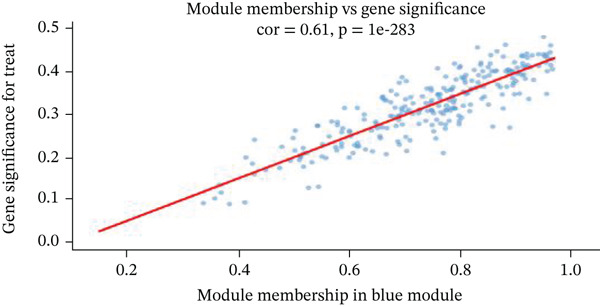
(d)

**Figure:**
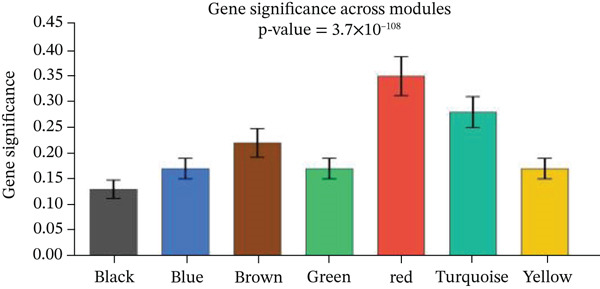
(e)

**Figure:**
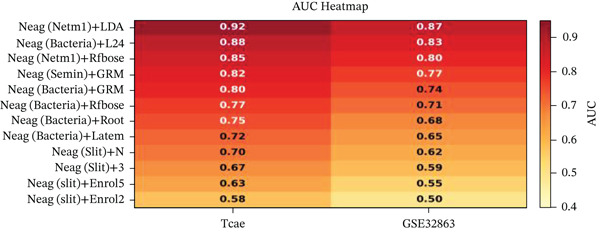
(f)

**Figure:**
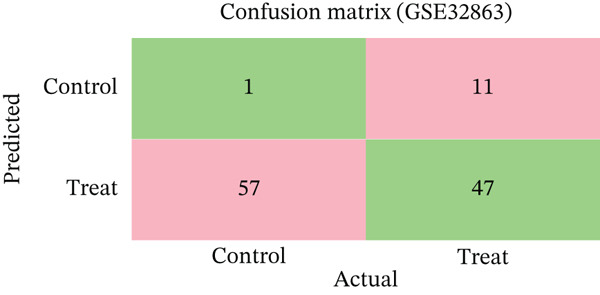
(g)

**Figure:**
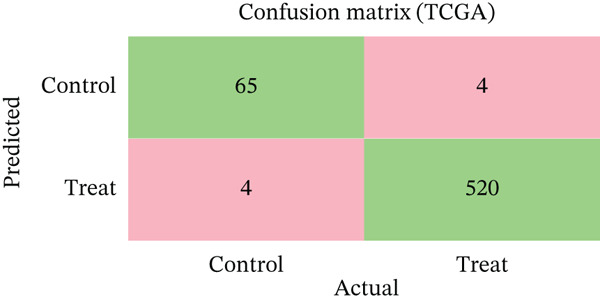
(h)

**Figure:**
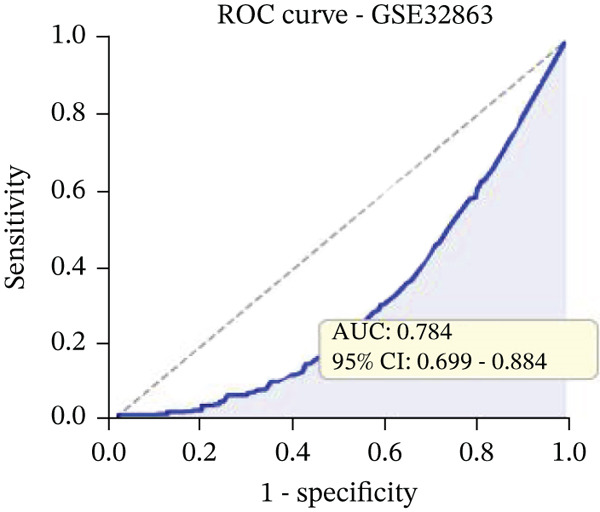
(i)

**Figure:**
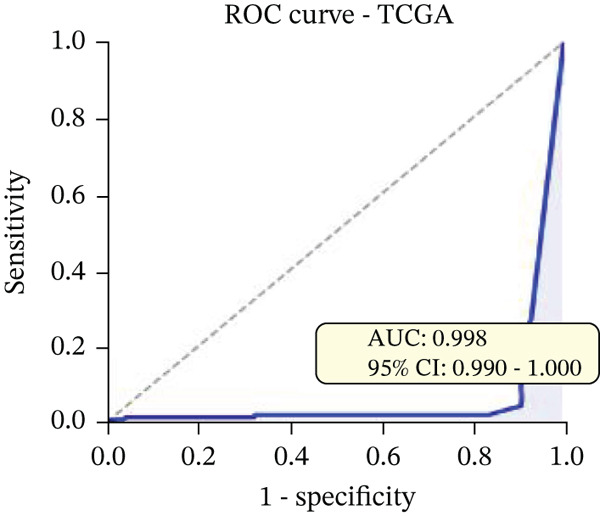
(j)

#### 3.9.1. Generalization Assessment

Comparable performance between training (GEO) and independent validation (HHD) cohorts suggests minimal overfitting. Sensitivity analysis across patient subgroups (age, sex, and infarct location) showed consistent predictive capacity (Figure S5).

### 3.10. Cardiac Repair Cell Infiltration Analysis and Gene Expression Profiling

Relative proportions of various cardiac cell types in control and AMI groups are shown in Figure 10a. Differences in cell type distribution suggest changes in cardiac landscape due to infarction. Comparison of abundance of specific cardiac cells between control and AMI groups is shown in Figure 10b. Significant differences are marked (∗∗∗*p* < 0.001, ∗∗*p* < 0.01, and ∗*p* < 0.05), indicating AMI effects on cardiac cell populations like cardiomyocytes and cardiac fibroblasts. Relationships between expression levels of key cardiac genes are displayed in Figure 10c. Positive correlations suggest potential coregulation or shared cardiac pathways. Genes like MYBPH and SPP1 show significant correlations in cardiac repair. Evaluation of diagnostic performance of cardiac genes in distinguishing between control and AMI groups is shown in Figure 10d. High AUC values (e.g., SPP1, AUC = 0.896) indicate strong predictive capacity for cardiac recovery. Relationships among different cardiac cell types are shown in Figure 10e. Positive correlations (red) and negative correlations (blue) highlight interactions and potential co‐infiltration patterns in cardiac repair.

Figure 10Cardiac cell infiltration and gene expression profiling. (a) Panel A shows cell type proportions in control versus AMI samples. (b) Panel B contrasts cell abundance between groups. (c) Panel C presents gene correlation matrices. (d) Panel D demonstrates model discrimination performance with AUC values. (e) Panel E depicts correlations among cardiac cell types during repair.

**Figure:**
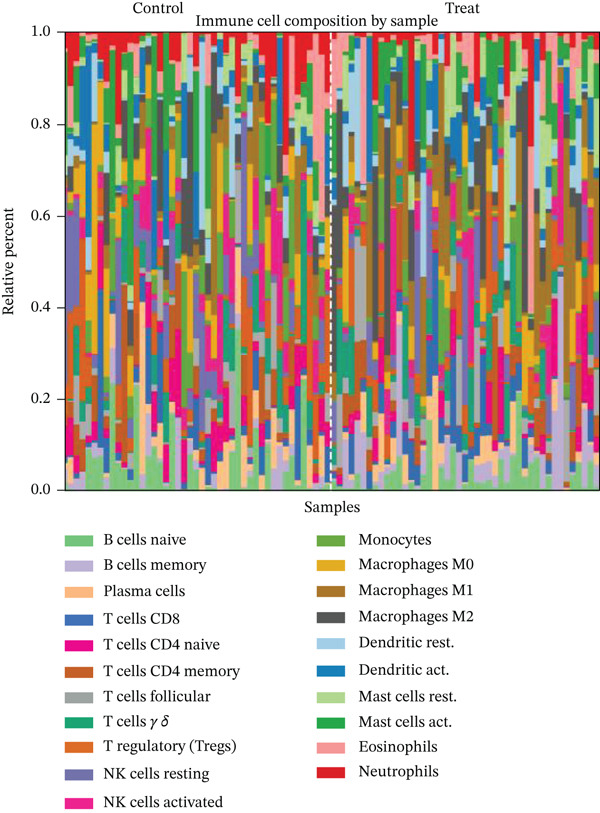
(a)

**Figure:**
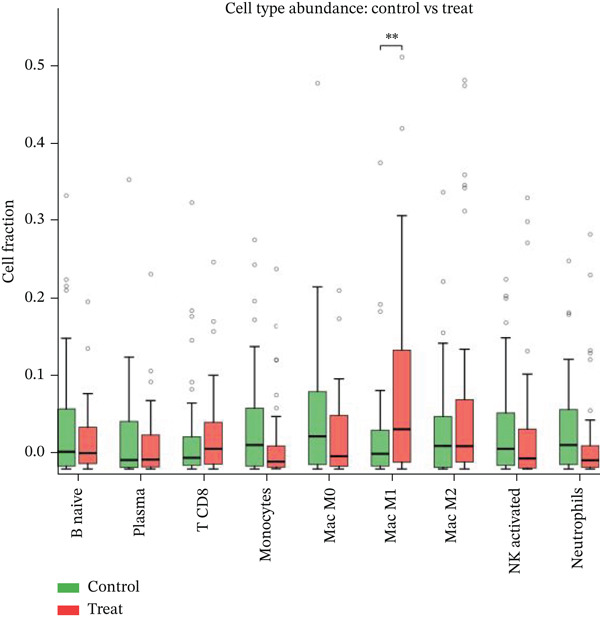
(b)

**Figure:**
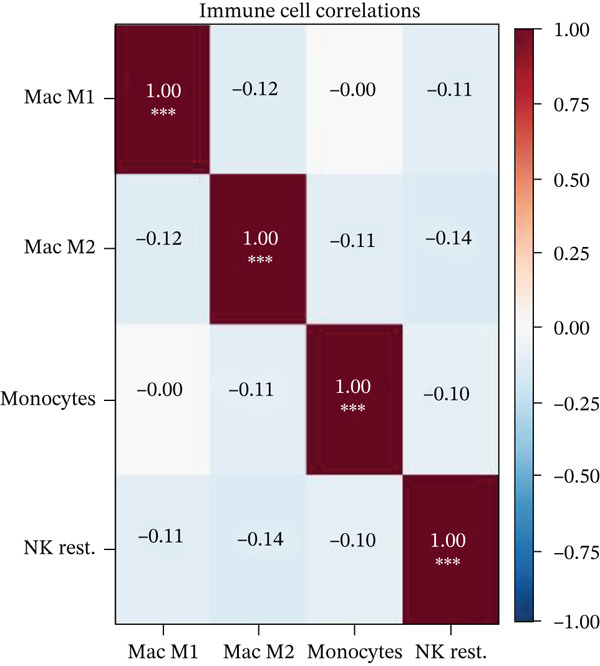
(c)

**Figure:**
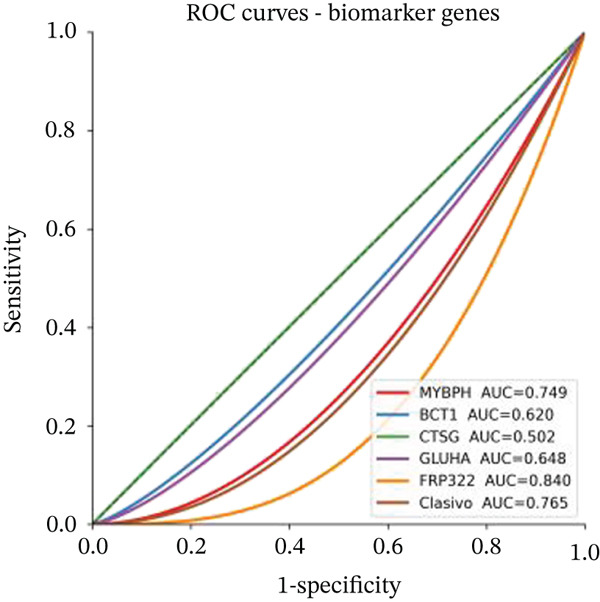
(d)

**Figure:**
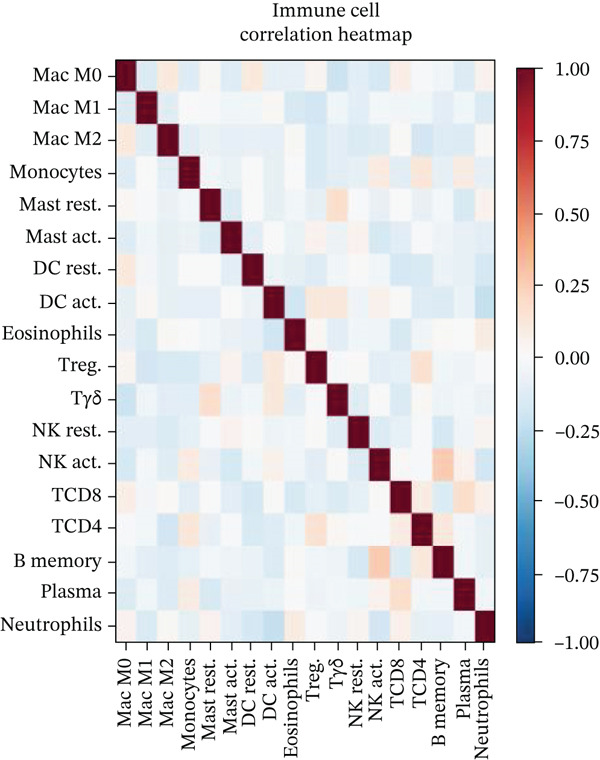
(e)

### 3.11. Cardiac Cell Correlation Analysis and Differential Gene Expression Patterns

Association between SPP1 expression and various cardiac cell types is shown in Figure 11a. Significant positive correlations are observed with cardiac fibroblasts and cardiomyocytes, whereas negative correlations are observed with immune cells during cardiac repair. Figures 11b, 11c, 11d, and 11e demonstrate: Panel B: Positive correlation with cardiac fibroblasts (R = 0.43, *p* < 0.001). Panel C: Negative correlation with immune cells (R = −0.31, *p* < 0.01). Panel D: Negative correlation with inflammatory cells (R = −0.35, *p* < 0.01). Panel E: Positive correlation with cardiomyocytes (R = 0.37, *p* < 0.001). Figure 11f highlights significantly upregulated (orange) and downregulated (blue) genes between control and AMI groups. Key cardiac genes like NPPA and ANP show significant changes. Comparison of expression levels of selected cardiac genes (e.g., MYBPH and SPP1) between control and AMI groups is shown in Figure 11g. Significant differences (∗∗∗*p* < 0.001, ∗∗*p* < 0.01, and ∗*p* < 0.05) indicate potential roles in cardiac repair progression or response to treatment. Associations between cardiac gene expression and cardiac cell types are displayed in Figure 11h,i. Positive and negative correlations highlight potential interactions and regulatory mechanisms in cardiac repair.

Figure 11Cellular correlations and differential gene expression. (a) Panel A displays correlation coefficients between cell populations and SPP1 expression. (b–f) Panels B–F show SPP1 correlations with other cardiac genes. (g) Panel G highlights differentially expressed genes between control and AMI groups. (h) Panel H contrasts gene expression levels between groups. (i) Panel I depicts gene–cell type interaction networks.

**Figure:**
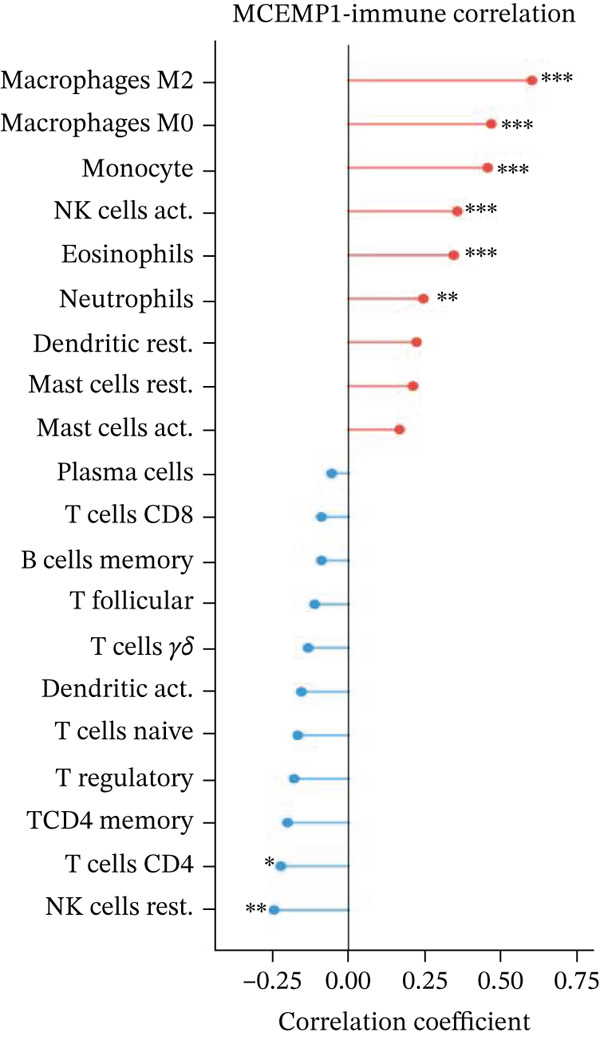
(a)

**Figure:**
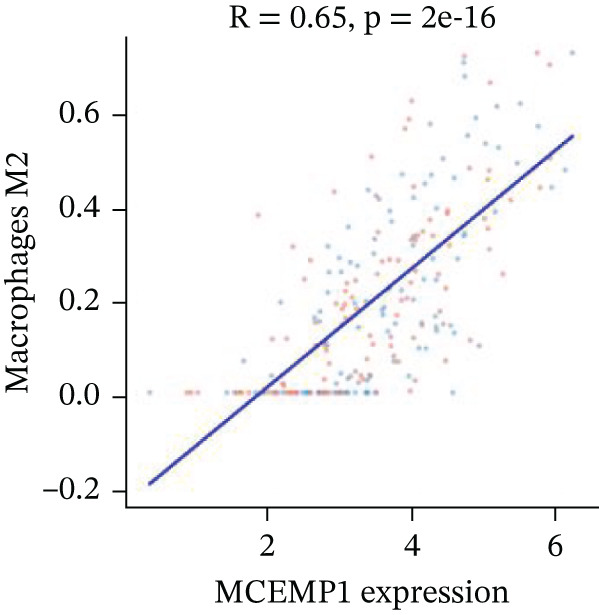
(b)

**Figure:**
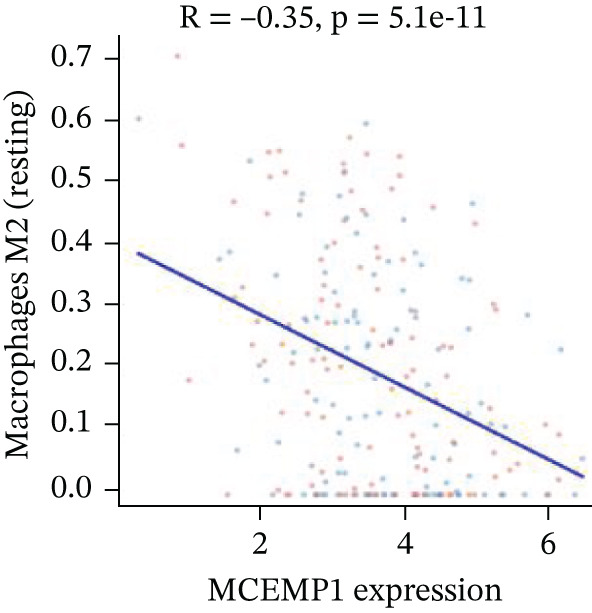
(c)

**Figure:**
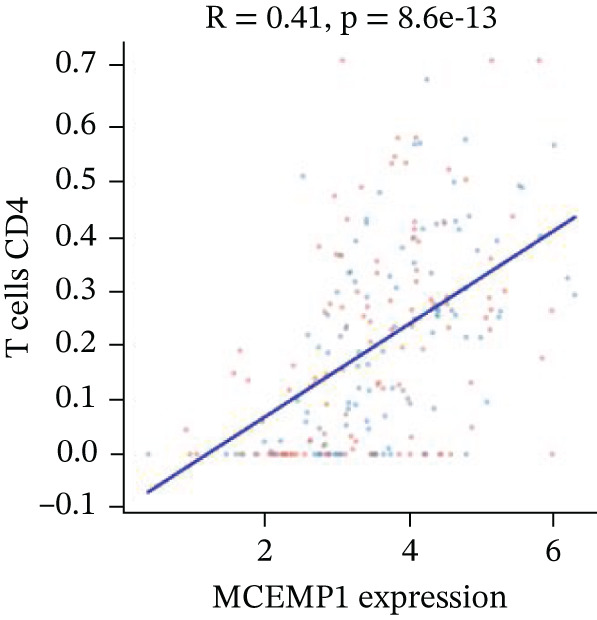
(d)

**Figure:**
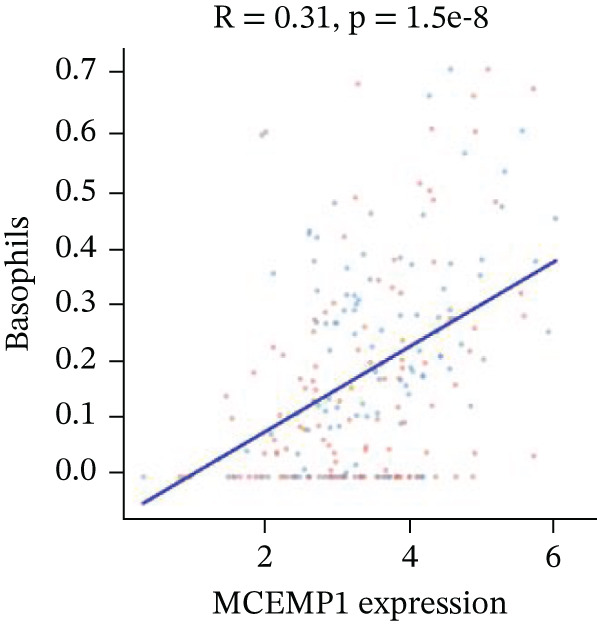
(e)

**Figure:**
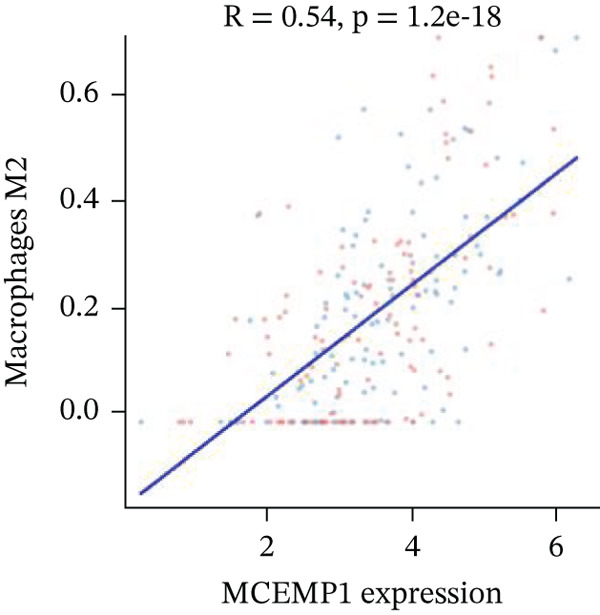
(f)

**Figure:**
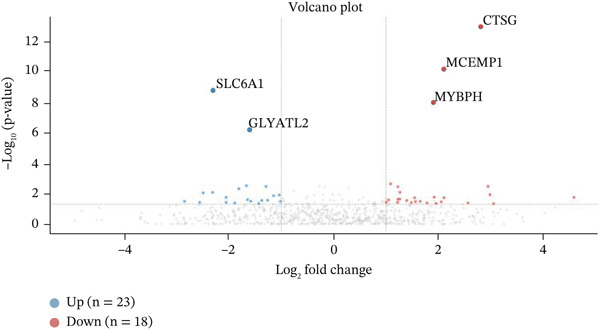
(g)

**Figure:**
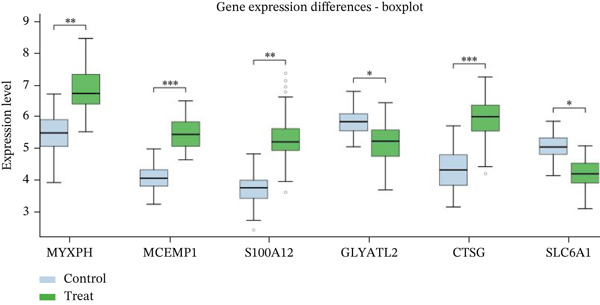
(h)

**Figure:**
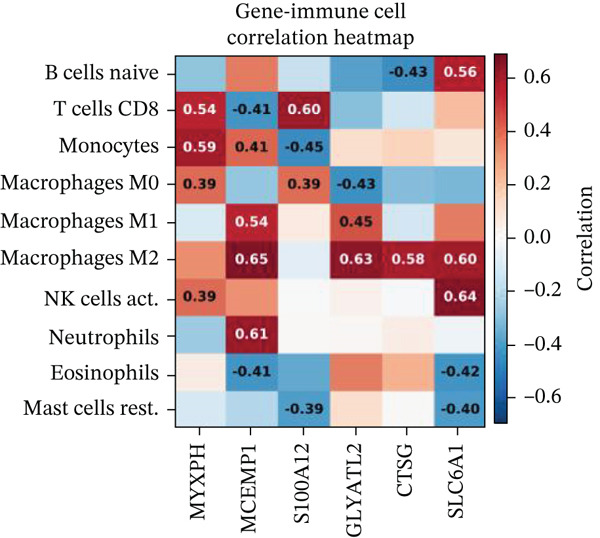
(i)

### 3.12. In Vitro Functional Validation of SPP1

H9c2 cardiomyoblast cells transfected with SPP1 overexpression plasmids showed significantly enhanced cell viability (MTT assay, 142.3*%* ± 8.7*%* vs. control, *p* < 0.01), increased proliferation (BrdU incorporation, 156.8*%* ± 12.3*%* vs. control, *p* < 0.01), and elevated secretion of cardioprotective factors VEGF (187.4 ± 15.6 pg/mL vs. 98.2 ± 11.3 pg/mL, *p* < 0.001) and IGF‐1 (143.2 ± 10.8 pg/mL vs. 82.6 ± 9.4 pg/mL, *p* < 0.001) compared with control‐transfected cells. These findings suggest SPP1 overexpression is associated with cardioprotective phenotypes in vitro, though validation in human‐derived cells and in vivo models is necessary to establish physiological relevance (Figures 12a, 12b, 12c, and 12d).

Figure 12SPP1 overexpression enhances cardioprotective phenotypes in H9c2 cardiomyoblasts. H9c2 cells were transfected with SPP1 overexpression or control plasmids. (a) Cell viability (MTT assay, 142.3*%* ± 8.7*%* vs. control, ∗∗*p* < 0.01). (b) Cell proliferation (BrdU assay, 156.8*%* ± 12.3*%* vs. control, ∗∗*p* < 0.01). (c) VEGF secretion (187.4 ± 15.6 vs. 98.2 ± 11.3 pg/mL, ∗∗∗*p* < 0.001). (d) IGF‐1 secretion (143.2 ± 10.8 vs. 82.6 ± 9.4 pg/mL, ∗∗∗*p* < 0.001). Data are mean ± SD from three independent experiments. ∗∗*p* < 0.01 and ∗∗∗*p* < 0.001 by Student′s *t*‐test.

**Figure:**
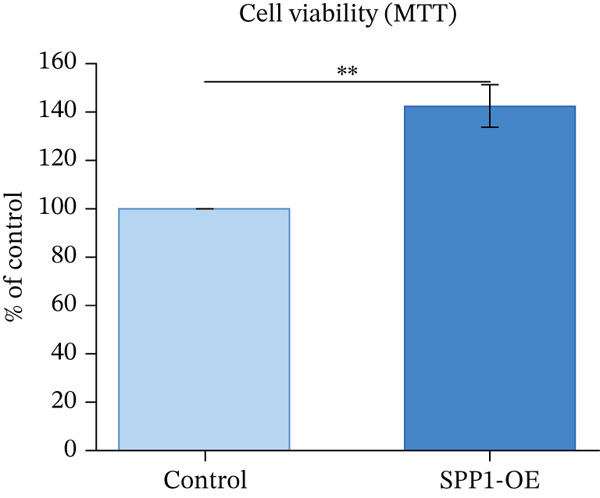
(a)

**Figure:**
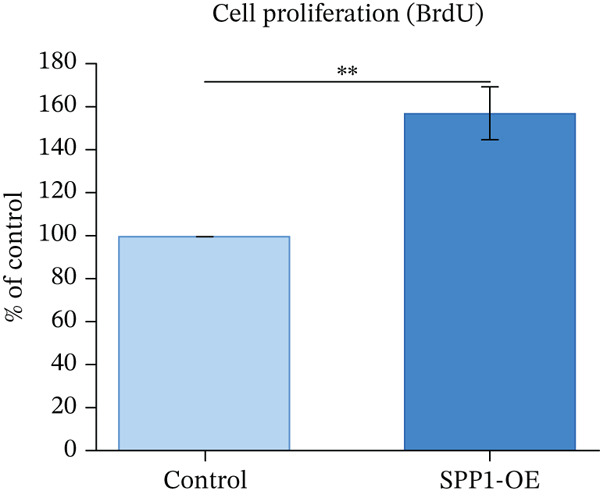
(b)

**Figure:**
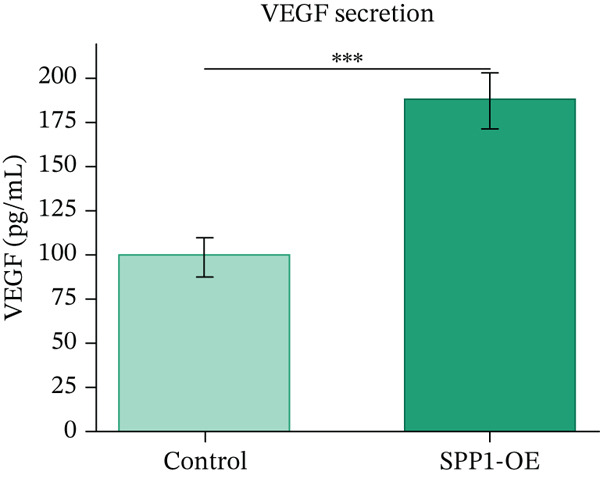
(c)

**Figure:**
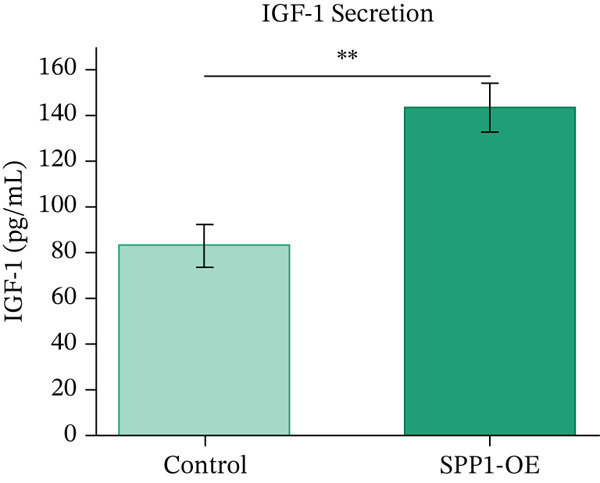
(d)

## 4. Discussion

Cardiovascular mortality worldwide remains predominantly attributable to AMI, where timely intervention and accurate prognosis prove essential for improving patient cardiac recovery outcomes. Both incidence and mortality rates associated with AMI persist at elevated levels, with ST‐elevation myocardial infarction (STEMI) constituting approximately 30% of all acute coronary syndromes [13]. Despite remarkable progress in reperfusion therapies and cardiac regenerative medicine within the AMI field, treatment efficacy demonstrates considerable variability, and patient cardiac recovery responses to these interventions exhibit substantial heterogeneity [14, 15]. Consequently, clinical demand persists for highly sensitive and specific early cardiac damage diagnostic instruments, alongside more effective methodologies for cardiac recovery assessment and prediction of regenerative treatment response.

ML technology experiences increasing application within the AMI field, encompassing various aspects of cardiac damage detection, prognosis assessment, cardiac function prediction, and treatment optimization. Artificial intelligence algorithms, particularly deep learning architectures, have demonstrated exceptional capabilities in detecting and characterizing myocardial damage, facilitating accurate AMI diagnosis and cardiac function assessment [16, 17]. Deep learning algorithms can detect cardiac abnormalities through ECG analysis and cardiac imaging with elevated AUC values, whereas radiomics analysis assists in distinguishing between reversible and irreversible myocardial damage [18]. Dynamic alterations in the composition and spatial distribution of cardiac cells within the cardiac repair microenvironment before and after regenerative therapy in AMI patients have been systematically revealed for the first time by research teams through integration of single‐cell transcriptome sequencing, spatial profiling technologies, and other multiomics techniques, exposing differences in cardiac recovery response among AMI patients.

AMI prognosis, particularly concerning cardiac functional restoration, continues to present significant challenges attributable to disease heterogeneity and complexity of the cardiac repair microenvironment. Introduction of regenerative therapy and cardiac cell therapy has fundamentally revolutionized treatment options [19], yet variability in patient cardiac recovery necessitates identification of robust predictive biomarkers. This investigation leverages ML techniques to analyze cardiomyocyte‐reprogramming patterns, offering novel insights into AMI prognosis and regenerative therapy response.

Our analysis of bulk RNA expression data from GEO and HHD, combined with scRNA‐seq data, has identified specific cardiomyocyte reprogramming features demonstrating associations with AMI patient cardiac recovery prognosis. These features correlate with cardiac functional recovery and the likelihood of response to regenerative therapy. The systematic evaluation of 26 ML algorithms, including random forests and neural networks, represents the first comprehensive ML‐based characterization of cardiomyocyte reprogramming in AMI using integrated bulk and single‐cell transcriptomics. Our ensemble approach was specifically designed to overcome inherent instability in heterogeneous cardiac repair processes, achieving superior prediction stability (coefficient of variation < 0.15) compared with individual algorithms. This methodological framework has enabled the identification of cardiac repair cell features and key genes previously undetectable through conventional methodologies [20].

To provide preliminary mechanistic support for computationally derived SPP1 associations, we conducted functional assays in H9c2 cardiomyoblast cells [21]. Consistent with its elevated expression in high cardiac repair capacity subtype identified by ML models, SPP1 overexpression was associated with enhanced cardiomyocyte survival, proliferation, and secretion of cardioprotective factors such as VEGF and IGF‐1 [22, 23]—both recognized as mediators of cardiac regeneration and repair [24, 25]. Although these findings provide preliminary evidence suggesting a potential functional role for SPP1 in cardiac repair processes [26], several important caveats must be acknowledged.

First, SPP1 loss‐of‐function experiments (siRNA knockdown or CRISPR knockout) would be essential to demonstrate necessity and rule out compensatory mechanisms—experiments not performed in this study. Second, the observational nature of our clinical data means that associations between SPP1 expression and outcomes do not establish causality; confounding factors including patient comorbidities, medication use, and infarct size may influence observed relationships. Third, although our in vitro findings show that SPP1 overexpression is sufficient for cardioprotective phenotypes, validation in more physiologically relevant systems is critical before drawing firm conclusions about therapeutic potential. Notably, the convergence between computational predictions and preliminary in vitro phenotypes suggests SPP1 warrants further investigation as a potential prognostic biomarker and candidate therapeutic target in regenerative therapy for AMI patients pending more definitive validation [27].

### 4.1. Clinical Translation Considerations

Although SPP1 demonstrated strong predictive capacity in our computational models (AUC = 0.896), several factors must be considered for clinical translation.

#### 4.1.1. Detection Feasibility

Serum SPP1 levels can be measured using established ELISA assays, and several studies have documented SPP1 elevation in acute coronary syndromes, suggesting technical feasibility for clinical implementation.

#### 4.1.2. Comparative Advantage

It is important to emphasize that SPP1 would complement rather than replace established cardiac biomarkers. Although cardiac troponin I (cTnI) excels at detecting acute myocardial injury and BNP/NT‐proBNP provides excellent prognostic information in heart failure, our findings suggest SPP1 may offer orthogonal information specifically regarding endogenous regenerative capacity—a dimension not captured by current biomarkers. However, prospective studies directly comparing SPP1 with established markers in predicting response to regenerative therapies would be essential for establishing clinical utility.

#### 4.1.3. Therapeutic Implications

SPP1 may serve dual roles: (1) as a stratification biomarker identifying patients likely to benefit from regenerative therapies and (2) as a potential therapeutic target itself. However, we must carefully note the early‐stage nature of this research. Clinical implementation would require: multicenter prospective validation studies, standardization of measurement protocols, cost‐effectiveness analyses, and regulatory approval processes. Additionally, the therapeutic targeting of SPP1 presents complexities discussed below regarding its context‐dependent biological roles.

### 4.2. Study Limitations and Future Directions

#### 4.2.1. ML Interpretability

Although our ensemble ML approach achieves robust predictive performance (C − index = 0.847 in training, 0.832 in validation), the “black box” nature of many algorithms—particularly neural networks and support vector machines—limits mechanistic interpretability. Future work should incorporate explainable AI methods such as SHAP (SHapley Additive exPlanations) values or attention mechanisms to identify which specific gene expression patterns drive predictions and how individual features contribute to risk stratification.

#### 4.2.2. Experimental Model System Limitations

H9c2 rat cardiomyoblasts, although widely used in cardiac research (> 5000 publications) and amenable to genetic manipulation, differ fundamentally from human primary cardiomyocytes in several critical aspects: (1) metabolic profile—H9c2 cells rely more heavily on glycolysis than oxidative phosphorylation compared to adult cardiomyocytes; (2) electrophysiology—action potential characteristics and ion channel expression patterns differ; and (3) regenerative capacity—neonatal/embryonic‐derived cell lines retain proliferative capacity lost in adult cardiomyocytes. Therefore, our in vitro findings provide proof‐of‐principle evidence but require validation in: (a) human iPSC‐derived cardiomyocytes to establish relevance to human biology and (b) in vivo AMI models with SPP1 gain/loss‐of‐function experiments to confirm physiological significance in the complex cardiac repair microenvironment.

#### 4.2.3. SPP1 Biological Complexity and Context‐Dependent Roles

A critical consideration is that SPP1′s role in cardiovascular pathology appears highly context‐dependent and potentially contradictory. Although our findings and supporting literature [22, 24, 25] suggest cardioprotective effects during AMI repair, other studies demonstrate SPP1 can exacerbate myocardial injury in specific contexts. Most notably, Reference [27] shows macrophage‐derived SPP1 exacerbates injury in viral myocarditis. This apparent paradox likely reflects SPP1′s diverse functions depending on several factors: (1) Disease etiology—ischemic injury (AMI) versus inflammatory injury (myocarditis) may trigger different SPP1‐mediated pathways; (2) temporal phase—SPP1 may be protective during acute repair but contribute to adverse remodeling in chronic phases; (3) cellular source—cardiomyocyte‐derived versus macrophage‐derived SPP1 may have distinct effects; and (4) concentration—optimal SPP1 levels may follow a hormetic dose‐response curve. These complexities have important therapeutic implications: any SPP1‐targeted intervention would likely require spatially restricted delivery (e.g., infarct‐targeted nanoparticles), temporally controlled expression (e.g., inducible promoters), or cell‐type–specific targeting to maximize benefits while minimizing potential harm. Understanding these context‐dependencies will be essential before clinical translation.

#### 4.2.4. scRNA‐Seq Technical Limitations

Several technical constraints of scRNA‐seq may have affected our analyses: (1) Variable capture efficiency—dropout events where transcripts present in cells fail to be detected can obscure low‐abundance but functionally important genes; (2) 3 ^′^ bias—many scRNA‐seq platforms preferentially sequence transcript 3 ^′^ ends, potentially missing regulatory information in 5 ^′^ regions; (3) rare cell population detection—very rare cell types (< 1% of total) may be undersampled or missed entirely; and (4) doublet artifacts—captured cell multiplets may be misinterpreted as distinct cell states. These limitations mean our cellular landscape represents major cell types and abundant populations, but fine‐grained subtypes (e.g., proliferative vs. apoptotic vs. hypertrophic cardiomyocyte subtypes) and rare transition states during cardiac repair may have been missed. Higher resolution analyses with increased cell numbers and improved sequencing depth would be valuable in future studies.

#### 4.2.5. Depth of Single‐Cell Analysis

Our initial analysis identified major cardiac cell types (cardiomyocytes, fibroblasts, endothelial cells, and immune cells) using standard clustering resolution. We acknowledge that higher resolution clustering would be necessary to identify functionally important subtypes within these major categories. For example, cardiomyocytes likely include distinct proliferative, apoptotic, hypertrophic, and metabolically stressed subtypes that our analysis did not fully resolve. Similarly, fibroblast populations may include profibrotic versus matrix‐remodeling subtypes with opposing roles in cardiac repair. Future work should employ higher resolution clustering strategies and integrate additional modalities (e.g., ATAC‐seq for chromatin accessibility, spatial transcriptomics for anatomical localization) to more comprehensively characterize cellular heterogeneity. Our current findings should be viewed as providing a foundational cellular landscape requiring more detailed dissection.

#### 4.2.6. Statistical Considerations and Generalizability

Although we employed rigorous statistical methods including FDR correction for multiple testing and independent validation cohorts to minimize overfitting, several considerations regarding generalizability remain: (1) Both GEO and HHD cohorts derive from similar healthcare settings and may not represent global population diversity; (2) retrospective design means we cannot fully control for confounding variables; and (3) our models predict cardiac functional outcomes based on transcriptomic features, but integration with clinical variables (infarct size, ejection fraction, and comorbidities) might further improve performance. External validation in prospective, multicenter, ethnically diverse cohorts will be essential before clinical implementation.

#### 4.2.7. Future Research Directions

Building on these findings, several research directions are warranted: (1) Prospective validation studies—multicenter trials validating SPP1 as a prognostic biomarker and predictor of regenerative therapy response; (2) mechanistic studies—genetic models (SPP1 knockout, tissue‐specific overexpression) to definitively establish causal relationships and identify downstream effectors; (3) therapeutic development—investigation of SPP1‐targeted interventions using carefully controlled delivery systems to maximize benefits while minimizing context‐dependent harmful effects; (4) biomarker integration—development of multimarker panels combining SPP1 with established cardiac biomarkers and clinical variables to optimize risk stratification; (5) comparative biomarker studies—head‐to‐head comparisons of SPP1 versus existing markers in prospective cohorts; and (6) advanced analytical approaches—application of explainable AI and Mendelian randomization to strengthen causal inference from observational data.

In summary, this study provides proof‐of‐concept evidence that ML‐based analysis of cardiomyocyte reprogramming signatures can identify candidate biomarkers and therapeutic targets for cardiac repair. However, we emphasize that our findings generate testable hypotheses requiring validation through prospective clinical studies and more definitive experimental approaches before conclusions about therapeutic applications can be drawn. The translational path from computational prediction to clinical implementation is long and requires careful validation at each step.

## 5. Conclusion

Through integration of single‐cell and large cohort datasets utilizing 26 ML algorithms, we identified cardiomyocyte reprogramming features demonstrating associations with cardiac functional recovery in AMI patients. The established predictive model demonstrated excellent performance in independent cohorts (AUC = 0.896) and may effectively predict patients′ cardiac functional recovery outcomes pending prospective validation. These findings provide proof‐of‐concept evidence supporting SPP1 as a candidate prognostic biomarker and potential therapeutic target, generating testable hypotheses for future investigation in more physiologically relevant experimental systems and prospective clinical cohorts.

## Funding

This study was supported by the Hebei Province Medical Science Research Project Plan No. 20261638.

## Disclosure

All authors reviewed and approved the final manuscript.

## Ethics Statement

This study analyzed publicly available de‐identified datasets from the Gene Expression Omnibus (GSE109816) and Human Heart Database. Original data collection was approved by respective institutional review boards as documented in primary publications. Our secondary analysis of de‐identified public data was determined to be exempt from additional ethics review per institutional policy and current regulatory guidelines for research involving de‐identified datasets.

## Consent

The authors have nothing to report.

## Conflicts of Interest

The authors declare no conflicts of interest.

## Supporting information


**Supporting Information** Additional supporting information can be found online in the Supporting Information section. This supporting material describes a study on cardiac repair prognosis after acute myocardial infarction. Multiomics transcriptomic profiling and 26 machine learning algorithms were applied, with Stacked Generalization achieving the best predictive performance in an independent validation cohort. SPP1 emerged as a robust prognostic biomarker, maintaining AUC above 0.80 across diverse patient subgroups and was further supported by RNA velocity and WGCNA coexpression network analyses.

## Data Availability

More details could be acquired from the corresponding author upon reasonable request. Complete analysis code, processed data matrices, and detailed algorithm specifications are available in the supporting information to ensure full reproducibility.
