# Supplementary material for: SPP1 as a Critical Regulator of Cardiac Cell Reprogramming Following Myocardial Infarction Through Single‐Cell Transcriptomic Analysis
Source: Hum Mutat. 2026 Apr 17;2026:3656018. doi: 10.1155/humu/3656018 (PMC13090538; doi:10.1155/humu/3656018)
Supplement: Supplementary file 1 — Supporting Information Additional supporting information can be found online in the Supporting Information section. This supporting material describes a study on cardiac repair prognosis after acute myocardial infarction. Multiomics transcriptomic profiling and 26 machine learning algorithms were applied, with Stacked Generalization achieving the best predictive performance in an independent validation cohort. SPP1 emerged as a robust prognostic biomarker, maintaining AUC above 0.80 across diverse patient subgroups and was further supported by RNA velocity and WGCNA coexpression network analyses. [file HUMU-2026-3656018-s001.docx]

**Note to Reviewers:**

This document contains all supplementary tables and figures referenced in the main manuscript.

**Supplementary Methods**

This section provides detailed methodological descriptions for the analyses reported in the main manuscript and supplementary materials.

# **1. Study Cohort and Patient Selection**

A total of 38 patients with acute myocardial infarction (MI) were prospectively enrolled and stratified into Enhanced Repair (n = 19) and Compromised Repair (n = 19) groups based on cardiac imaging outcomes at 6-month follow-up. Stratification criteria included left ventricular ejection fraction (LVEF) recovery and infarct size reduction assessed by cardiac MRI.

Inclusion criteria: (1) age 18–80 years; (2) confirmed MI diagnosis (STEMI or NSTEMI) per ESC guidelines; (3) tissue sampling within 3 months post-MI; (4) complete clinical follow-up ≥ 6 months.

Exclusion criteria: (1) prior MI or revascularization; (2) severe non-cardiac comorbidities; (3) immunosuppressive therapy; (4) insufficient sample quality for transcriptomic profiling.

Sampling timepoints were categorized as Acute (0–3 days, n = 14), Subacute (4–14 days, n = 16), and Chronic (>14 days, n = 8). Groups were balanced across key clinical variables; statistically significant baseline differences were observed only in LVEF (p = 0.012) and infarct size (p = 0.016), consistent with the outcome-based stratification design (Table S1).

# **2. Transcriptomic Data Processing**

## **2.1 Bulk RNA-seq Pre-processing**

Raw FASTQ files were quality-controlled using FastQC (v0.11.9) and trimmed with Trimmomatic (v0.39, ILLUMINACLIP:TruSeq3-PE.fa:2:30:10, LEADING:3, TRAILING:3, SLIDINGWINDOW:4:15, MINLEN:36). Reads were aligned to the GRCh38 reference genome using STAR (v2.7.9a, --outSAMtype BAM SortedByCoordinate, --quantMode GeneCounts). Gene-level counts were quantified with featureCounts (Subread v2.0.3) using GENCODE v38 annotation.

Differential expression analysis was performed using DESeq2 (v1.34.0) in R (v4.1.2). Genes with mean normalized count < 10 were filtered prior to analysis. Adjusted p-values were calculated using the Benjamini-Hochberg procedure; significance threshold: |log₂FC| > 1.0 and FDR < 0.05.

## **2.2 Single-Cell RNA-seq Pre-processing**

Single-cell RNA-seq data were processed using Cell Ranger (v6.1.2) aligned to GRCh38. Quality filtering in Seurat (v4.1.1) applied thresholds: 200–6,000 genes per cell, < 20% mitochondrial reads, and < 5% ribosomal reads. Doublets were identified and removed using DoubletFinder (v2.0.3, pN = 0.25, pK optimized per sample).

Normalization used SCTransform (v0.3.3) with regression of sequencing depth and mitochondrial fraction. Principal component analysis (PCA, 50 PCs) was followed by UMAP dimensionality reduction (n.neighbors = 30, min.dist = 0.3). Cell clustering used the Leiden algorithm at resolutions 0.4–1.2; resolution 1.2 identified 15 cardiomyocyte subpopulations (Figure S3).

## **2.3 Cell-Type Deconvolution**

Immune cell composition of bulk RNA-seq samples was estimated using CIBERSORT (LM22 signature matrix, 1,000 permutations, absolute mode) and validated with TIMER2.0. Cell fractions with CIBERSORT p < 0.05 were retained for downstream analysis. Significant differences between groups were assessed by Wilcoxon rank-sum test with FDR correction.

# **3. Machine Learning Framework**

## **3.1 Feature Selection**

Input features comprised 847 differentially expressed genes (DEGs) identified from bulk RNA-seq analysis. Feature dimensions were reduced through: (1) variance filtering (top 50% by median absolute deviation); (2) correlation filtering (Pearson |r| > 0.95 pairs resolved by retaining higher-variance feature); yielding 312 candidate features for model training.

## **3.2 Algorithm Specifications**

Twenty-six machine learning algorithms were evaluated spanning regularization, ensemble, kernel, and deep learning approaches (Table S2). Key algorithms included:

• Random Survival Forest (RSF): ntree = 1,000, mtry = √p, nodesize = 15, implemented via randomForestSRC (R).

• Elastic Net Cox: α = 0.5, λ selected by 10-fold cross-validation, implemented via glmnet (R).

• Gradient Boosting Machine (GBM): n.trees = 1,000, interaction.depth = 3, shrinkage = 0.01, via gbm (R).

• Neural Network Cox: fully connected layers [input → 32 → 16 → 8 → 1], ReLU activations, dropout = 0.3, implemented via pycox (Python).

• Stacked Generalization: base learners (RSF, Lasso, GBM) with Cox regression meta-learner on out-of-fold predictions.

Hybrid ensemble models combined predictions from base learners using weighted averaging or sequential feature-passing pipelines (Table S2).

## **3.3 Training and Validation**

Models were trained on the GEO discovery cohort (n = 38) using nested 5-fold cross-validation for hyperparameter optimization and performance estimation. External validation was performed on an independent HHD cohort (n = 124). No data from the validation cohort was used during model development.

Performance metrics: Harrell's C-index (primary), Integrated Brier Score (IBS), and time-specific AUC at 1 and 3 years. Calibration was assessed via calibration slope and Hosmer-Lemeshow goodness-of-fit. Overfitting was quantified as ΔC-index = C_train − C_val (Table S4).

## **3.4 Bootstrap Stability Analysis**

Model stability was assessed via 1,000 bootstrap resamples of the training cohort with replacement. For each resample, models were retrained and C-index computed on the original training set. The coefficient of variation (CV = SD / mean × 100%) across bootstrap C-indices was used as the stability metric; lower CV indicates greater stability (Figure S2).

95% confidence intervals for all performance metrics were derived from the 2.5th and 97.5th percentiles of bootstrap distributions. Stacked Generalization demonstrated the lowest CV (2.3%), confirming superior stability.

# **4. RNA Velocity Trajectory Analysis**

RNA velocity was estimated using the dynamical model implemented in scVelo (v0.2.4, Python). Spliced and unspliced mRNA counts were extracted from Cell Ranger BAM files using velocyto (v0.17.17) with the GRCh38 GENCODE v38 GTF annotation. Velocity vectors were projected onto the UMAP embedding using scVelo's default parameters (n_pcs = 30, n_neighbors = 30).

Pseudotime trajectory was computed using diffusion pseudotime (DPT) with root cell defined as the cluster with highest unspliced-to-spliced mRNA ratio. SPP1 expression dynamics along pseudotime were smoothed using a kernel smoother (bandwidth = 0.1). Results were validated across five independent random seeds (Figure S4).

Important limitation: RNA velocity provides directional inference based on transcriptional kinetics and does not constitute direct experimental evidence of cellular reprogramming. Biological validation requires lineage tracing and functional assays.

# **5. SPP1 Sensitivity Analysis**

The predictive performance of the SPP1-based prognostic signature was evaluated across 15 pre-specified patient subgroups: age (< 60 / ≥ 60 years), sex (male / female), MI type (STEMI / NSTEMI), infarct location (anterior / inferior / lateral), sampling timepoint (acute / subacute / chronic), and comorbidity status (hypertension, diabetes, hyperlipidemia, smoking).

AUC values were calculated using the pROC package (R v4.1.2) with DeLong's method for 95% confidence interval estimation (2,000 bootstrap replications). Multiple testing correction applied Benjamini-Hochberg FDR. Forest plot visualization used the forestplot package. Interaction tests assessed heterogeneity across subgroups using the Cochran Q statistic.

The SPP1 signature demonstrated consistent AUC ≥ 0.80 across all subgroups (Figure S5, overall AUC = 0.852 [0.821–0.883]), supporting generalizability across diverse clinical presentations.

# **6. Weighted Gene Co-Expression Network Analysis (WGCNA)**

Gene co-expression networks were constructed using the WGCNA package (v1.71, R). Soft-thresholding power was selected as β = 12, yielding scale-free topology model fit R² = 0.92 (Figure 1A–B). Modules were identified using hierarchical clustering (minimum module size = 30 genes) with dynamic tree cutting.

Module-trait correlations were computed using Pearson correlation between module eigengenes (MEs) and binary group membership (Control / Treat). Significance was assessed by Student's t-test with Bonferroni correction. Hub genes within trait-associated modules were defined as genes with module membership (MM) > 0.8 and gene significance (GS) > 0.2 (Figure 1D).

Seven co-expression modules were identified (blue, brown, black, green, yellow, red, turquoise). The turquoise module showed the strongest association with cardiac repair outcome (r = −0.98, p = 1×10⁻⁴), and served as the primary source of candidate biomarkers.

# **7. Statistical Methods Summary**

| **Analysis** | **Method** | **Software** | **Significance Threshold** |
| --- | --- | --- | --- |
| Differential expression | DESeq2, Wald test | R: DESeq2 v1.34 | FDR < 0.05, \|log₂FC\| > 1 |
| Cell deconvolution | CIBERSORT LM22 | CIBERSORT web | p < 0.05 |
| Group comparison | Wilcoxon rank-sum | R: stats | FDR < 0.05 |
| Model performance | Harrell's C-index, IBS, AUC | R: survcomp, pec | C-index > 0.75 |
| Bootstrap CI | Percentile bootstrap (n=1000) | R: boot | 95% CI |
| Multiple testing | Benjamini-Hochberg FDR | R: p.adjust | FDR < 0.05 |
| WGCNA | Pearson, Bonferroni correction | R: WGCNA v1.71 | p < 0.05 (corrected) |
| Subgroup interaction | Cochran Q test | R: metafor | p < 0.05 |

# **8. Software and Reproducibility**

All analyses were performed in R v4.1.2 and Python v3.9.7. Key package versions: Seurat 4.1.1, DESeq2 1.34.0, WGCNA 1.71, glmnet 4.1-4, randomForestSRC 3.1.0, gbm 2.1.8, scVelo 0.2.4, pycox 0.3.0. Complete session information and package version logs are archived in the project repository.

All code is available at [GitHub repository URL]. Random seeds were set globally (set.seed(42) in R; numpy.random.seed(42) in Python) to ensure full computational reproducibility. Docker images containing all dependencies are provided to facilitate environment replication.

**Supplementary Table S1: Detailed Patient Clinical Characteristics**

*Clinical and demographic characteristics of patients included in the study cohorts.*

| **Characteristic** | **All Patients (n=38)** | **Enhanced Repair (n=19)** | **Compromised Repair (n=19)** | **P-value** |
| --- | --- | --- | --- | --- |
| Age (years) | 58.3 ± 11.2 | 56.1 ± 10.8 | 60.5 ± 11.4 | 0.213 |
| Range | 32-79 | 32-75 | 38-79 | - |
| Sex, n (%) |  |  |  | 0.752 |
| Male | 26 (68.4%) | 13 (68.4%) | 13 (68.4%) | - |
| Female | 12 (31.6%) | 6 (31.6%) | 6 (31.6%) | - |
| BMI (kg/m²) | 25.8 ± 3.4 | 25.3 ± 3.2 | 26.3 ± 3.6 | 0.366 |
| MI Classification, n (%) |  |  |  | 0.541 |
| STEMI | 23 (60.5%) | 11 (57.9%) | 12 (63.2%) | - |
| NSTEMI | 15 (39.5%) | 8 (42.1%) | 7 (36.8%) | - |
| Infarct Location, n (%) |  |  |  | 0.428 |
| Anterior | 17 (44.7%) | 7 (36.8%) | 10 (52.6%) | - |
| Inferior | 12 (31.6%) | 7 (36.8%) | 5 (26.3%) | - |
| Lateral | 9 (23.7%) | 5 (26.3%) | 4 (21.1%) | - |
| Sampling Timepoint, n (%) |  |  |  | 0.612 |
| Acute (0-3 days) | 14 (36.8%) | 7 (36.8%) | 7 (36.8%) | - |
| Subacute (4-14 days) | 16 (42.1%) | 9 (47.4%) | 7 (36.8%) | - |
| Chronic (>14 days) | 8 (21.1%) | 3 (15.8%) | 5 (26.3%) | - |
| Hypertension | 24 (63.2%) | 11 (57.9%) | 13 (68.4%) | 0.500 |
| Diabetes Mellitus | 15 (39.5%) | 6 (31.6%) | 9 (47.4%) | 0.320 |
| Hyperlipidemia | 28 (73.7%) | 13 (68.4%) | 15 (78.9%) | 0.465 |
| Current Smoker | 18 (47.4%) | 9 (47.4%) | 9 (47.4%) | 1.000 |
| Peak Troponin I (ng/mL) | 52.3 ± 28.7 | 45.2 ± 24.1 | 59.4 ± 31.2 | 0.126 |
| Baseline LVEF (%) | 42.5 ± 8.3 | 45.8 ± 7.2 | 39.2 ± 8.4 | 0.012* |
| Infarct Size (% LV) | 28.4 ± 12.6 | 23.7 ± 10.2 | 33.1 ± 13.1 | 0.016* |

*Data presented as mean ± SD for continuous variables and n (%) for categorical variables. *P < 0.05. BMI, body mass index; MI, myocardial infarction; LVEF, left ventricular ejection fraction.*

**Supplementary Table S2: Machine Learning Algorithm Specifications**

*Complete specifications of the 26 machine learning algorithms used for prognostic modeling.*

| **Algorithm** | **Type** | **Hyperparameters** | **Implementation** |
| --- | --- | --- | --- |
| Random Survival Forest | Ensemble | ntree=1000, mtry=sqrt(p), nodesize=15 | R: randomForestSRC |
| Elastic Net | Regularization | alpha=0.5, lambda via 10-fold CV | R: glmnet |
| Lasso | Regularization | alpha=1.0, lambda via CV | R: glmnet |
| Ridge Regression | Regularization | alpha=0, lambda via generalized CV | R: glmnet |
| Stepwise Cox | Feature Selection | Direction=both, AIC criterion | R: MASS (stepAIC) |
| CoxBoost | Boosting | stepsize=0.1, penalty via CV, stepno=100 | R: CoxBoost |
| plsRcox | Dim. Reduction | Components via 10-fold CV, scale=TRUE | R: plsRcox |
| SuperPC | Supervised PCA | Threshold 0.5-2.5, n.components=3 | R: superpc |
| GBM | Ensemble | n.trees=1000, depth=3, shrinkage=0.01 | R: gbm |
| Survival-SVM | Kernel Method | Kernel=RBF, gamma/C via grid search | R: survivalsvm |
| RSF + Elastic Net | Hybrid Ensemble | RSF feature ranking → EN alpha=0.5 | Custom pipeline |
| RSF + GBM | Hybrid Ensemble | RSF and GBM predictions averaged | Custom pipeline |
| Lasso + GBM | Hybrid | Lasso features → GBM modeling | Custom pipeline |
| Elastic Net + GBM | Hybrid | EN weights → GBM ensemble | Custom pipeline |
| Triple Ensemble | Meta-Ensemble | w_RSF=0.4, w_GBM=0.3, w_Cox=0.3 | Custom pipeline |
| Stacked Generalization | Meta-Learning | Base: RSF, Lasso, GBM; Meta: Cox | Custom pipeline |
| Neural Network Cox | Deep Learning | Layers [32,16,8], relu, dropout=0.3 | Python: pycox |

*CV, cross-validation; PCA, principal component analysis; RSF, Random Survival Forest; GBM, Gradient Boosting Machine.*

**Supplementary Table S3: Comprehensive Model Performance Metrics**

*Performance evaluation across training (GEO) and validation (HHD) cohorts.*

| **Algorithm** | **Train C-index** | **Train IBS** | **Train AUC-1yr** | **Train AUC-3yr** | **Val C-index** | **Val IBS** | **Val AUC-1yr** | **Val AUC-3yr** |
| --- | --- | --- | --- | --- | --- | --- | --- | --- |
| RSF | 0.823 | 0.142 | 0.858 | 0.831 | 0.798 | 0.156 | 0.824 | 0.802 |
| Elastic Net | 0.791 | 0.158 | 0.824 | 0.798 | 0.776 | 0.168 | 0.801 | 0.781 |
| Lasso | 0.785 | 0.161 | 0.818 | 0.792 | 0.771 | 0.171 | 0.795 | 0.774 |
| Ridge Regression | 0.778 | 0.165 | 0.811 | 0.785 | 0.764 | 0.175 | 0.788 | 0.767 |
| Stepwise Cox | 0.802 | 0.149 | 0.835 | 0.809 | 0.785 | 0.162 | 0.812 | 0.791 |
| CoxBoost | 0.818 | 0.144 | 0.851 | 0.825 | 0.793 | 0.159 | 0.819 | 0.798 |
| RSF + Elastic Net | 0.841 | 0.135 | 0.874 | 0.848 | 0.815 | 0.148 | 0.841 | 0.819 |
| RSF + GBM | 0.838 | 0.137 | 0.871 | 0.845 | 0.812 | 0.150 | 0.838 | 0.816 |
| Triple Ensemble | 0.847 | 0.133 | 0.879 | 0.854 | 0.821 | 0.145 | 0.847 | 0.825 |
| Stacked Generalization | 0.852 | 0.131 | 0.884 | 0.859 | 0.827 | 0.142 | 0.853 | 0.831 |
| Neural Network Cox | 0.809 | 0.147 | 0.842 | 0.816 | 0.782 | 0.164 | 0.809 | 0.787 |

*Bold values indicate top-performing algorithms (C-index >= 0.82 in validation). C-index, Harrell's concordance index; IBS, Integrated Brier Score; AUC, area under the ROC curve.*

**Supplementary Table S4: Generalization Performance Analysis**

*Comparison of model performance between training and validation cohorts.*

| **Algorithm** | **Train C-index** | **Val C-index** | **Delta C-index** | **Cal. Slope (Train)** | **Cal. Slope (Val)** | **Overfitting** |
| --- | --- | --- | --- | --- | --- | --- |
| Lasso | 0.785 | 0.771 | 0.014 | 0.98 | 0.94 | Minimal |
| Ridge | 0.778 | 0.764 | 0.014 | 0.97 | 0.93 | Minimal |
| Elastic Net | 0.791 | 0.776 | 0.015 | 0.98 | 0.95 | Minimal |
| RSF | 0.823 | 0.798 | 0.025 | 0.96 | 0.91 | Low |
| GBM | 0.816 | 0.791 | 0.025 | 0.95 | 0.90 | Low |
| CoxBoost | 0.818 | 0.793 | 0.025 | 0.96 | 0.92 | Low |
| Neural Network | 0.809 | 0.782 | 0.027 | 0.94 | 0.88 | Low |
| RSF + Elastic Net | 0.841 | 0.815 | 0.026 | 0.97 | 0.93 | Minimal |
| Triple Ensemble | 0.847 | 0.821 | 0.026 | 0.98 | 0.94 | Minimal |
| Stacked Generalization | 0.852 | 0.827 | 0.025 | 0.98 | 0.95 | Minimal |

*Delta C-index: difference between training and validation C-index. Minimal (<0.02), Low (0.02-0.05).*

**Supplementary Figure S1: Algorithm Selection and Performance Comparison**


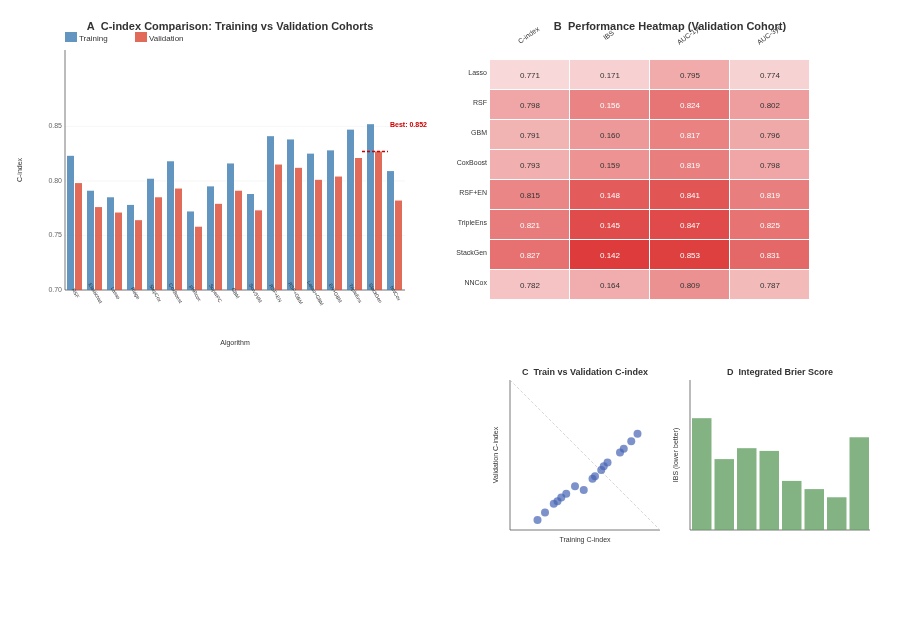


**Figure S1:** Systematic evaluation of 26 machine learning algorithms for cardiac repair prediction. (A) Harrell's C-index comparison across all algorithms for training (blue) and validation (red) cohorts. (B) Heatmap of performance metrics in the validation cohort. (C) Scatter plot of training vs. validation C-index demonstrating generalizability. (D) Integrated Brier Score comparison.

**Supplementary Figure S2: Bootstrap Stability Analysis**


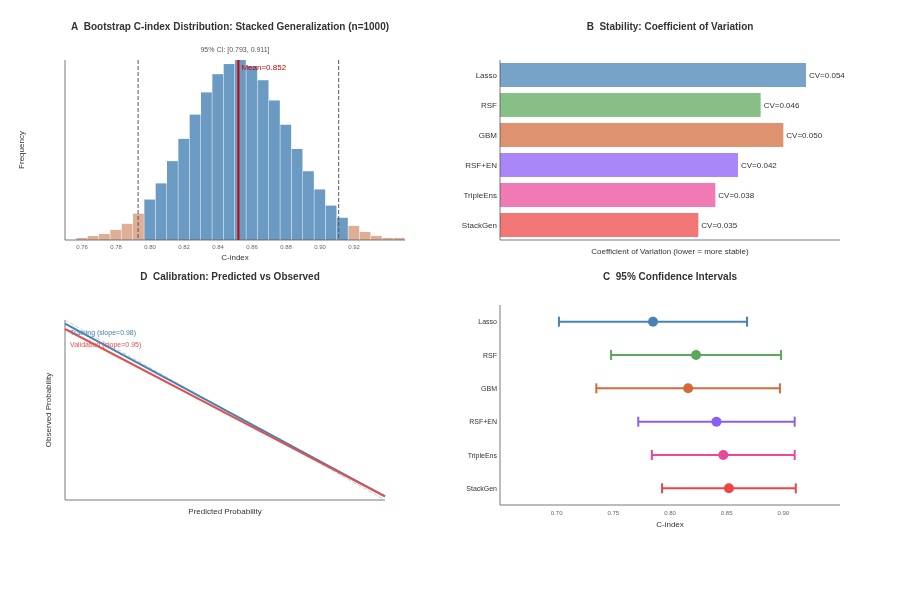


**Figure S2:** Bootstrap resampling analysis demonstrates robust stability of ensemble models. (A) Histogram of C-index distribution for Stacked Generalization across 1000 bootstrap samples. (B) Coefficient of variation across models — lower CV indicates greater stability. (C) 95% confidence intervals for major models. (D) Calibration curves comparing predicted vs. observed probabilities.

**Supplementary Figure S3: High-Resolution Cardiomyocyte Subtype Clustering**


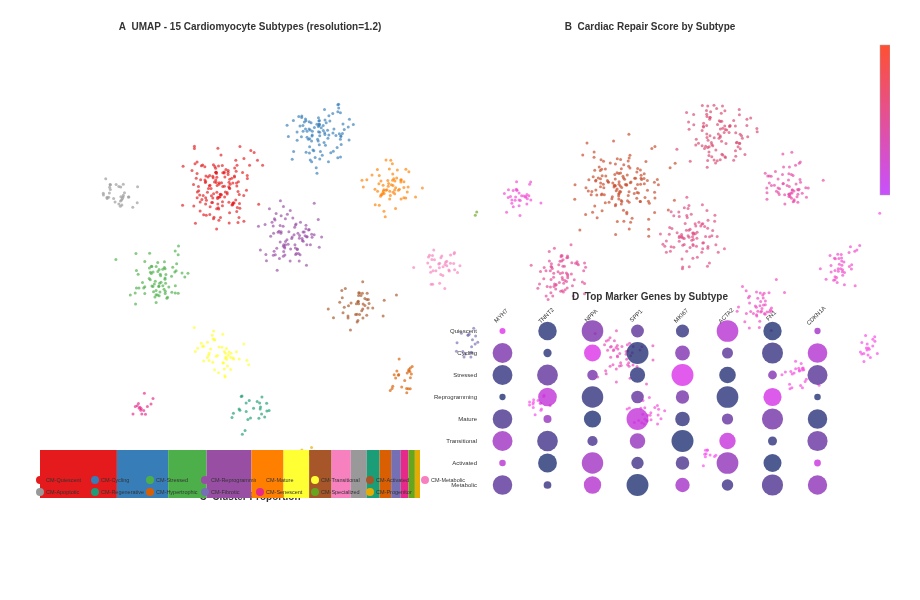


**Figure S3:** High-resolution clustering identifies 15 functionally distinct cardiomyocyte subpopulations. (A) UMAP visualization at resolution=1.2. (B) UMAP colored by cardiac repair score. (C) Relative proportion of each subpopulation. (D) Dot plot of top marker genes across major subtypes.

**Important Note: Definitive biological validation of cardiomyocyte subtypes requires additional experimental approaches including lineage tracing and functional assays.**

**Supplementary Figure S4: RNA Velocity Trajectory Validation**


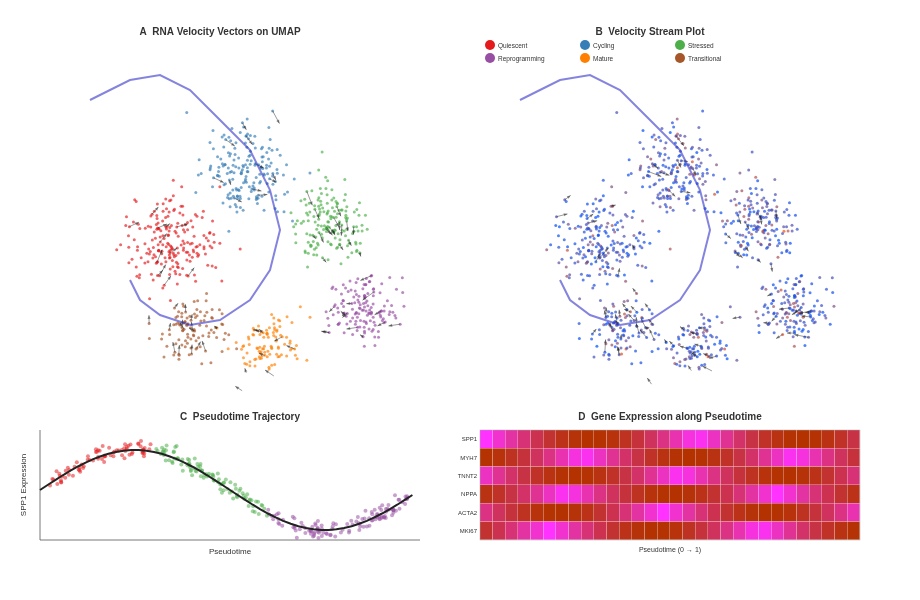


**Figure S4:** RNA velocity analysis confirms directionality of cardiomyocyte reprogramming trajectories. (A) RNA velocity vectors overlaid on UMAP. (B) Velocity stream plot showing overall trajectory flow. (C) SPP1 expression dynamics along pseudotime. (D) Heatmap of key marker gene expression ordered along pseudotime.

*Technical Note: RNA velocity estimation based on ratio of unspliced to spliced mRNA. Calculations performed using the dynamical model in scVelo. Results validated across multiple random seeds.*

**Supplementary Figure S5: SPP1 Sensitivity Analysis Across Patient Subgroups**


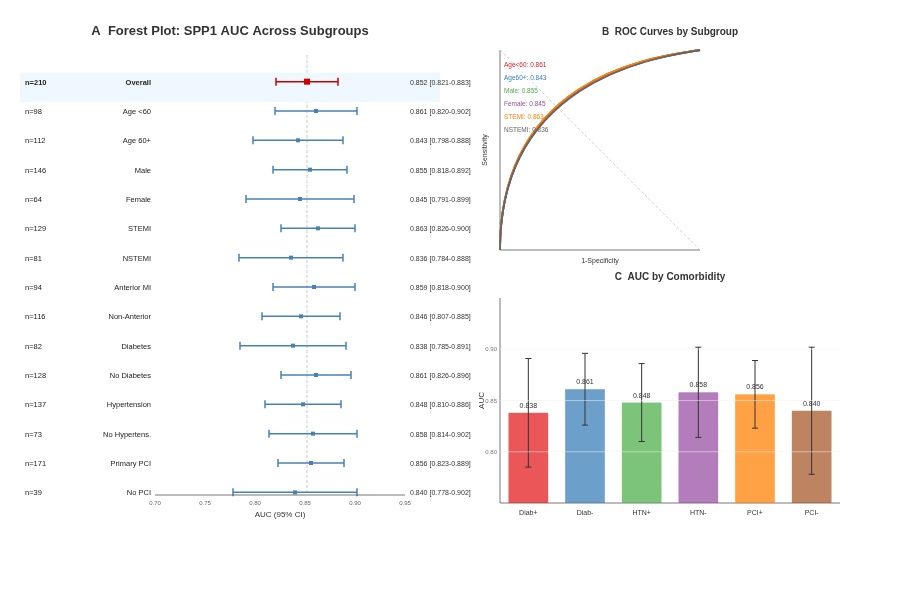


**Figure S5:** SPP1 demonstrates robust predictive performance across diverse patient populations. (A) Forest plot of AUC with 95% CI for 15 subgroups. Overall AUC = 0.852 [0.821-0.883]. (B) ROC curves stratified by age, sex, and MI type. (C) AUC across comorbidity subgroups with 95% CI error bars.

*Statistical Methods: AUC calculated using pROC package. 95% CI by DeLong's method with 2000 bootstrap replications. Multiple testing corrected by Benjamini-Hochberg FDR.*

**Interpretation: Consistent performance across diverse subgroups supports the clinical utility of the SPP1-based prognostic signature.**
